# Decoding Undesirable Inflammatory Responses of Nucleic Acid‐Delivering Lipid Nanoparticles

**DOI:** 10.1002/advs.75548

**Published:** 2026-05-07

**Authors:** Ruimin Hu, Yin Dou, Chenwen Li, Yuying Mu, Huiying Ouyang, Wenhao Shen, Jianxiang Zhang

**Affiliations:** ^1^ Department of Urology The First Affiliated Hospital (Southwest Hospital) Army Medical University (Third Military Medical University) Chongqing P. R. China; ^2^ Department of Pharmaceutics College of Pharmacy Third Military Medical University (Army Medical University) Chongqing P. R. China; ^3^ Yu‐Yue Pathology Scientific Research Center Chongqing P. R. China; ^4^ State Key Laboratory of Trauma and Chemical Poisoning Third Military Medical University (Army Medical University) Chongqing P. R. China; ^5^ The First Clinical College Chongqing Medical University Chongqing P. R. China; ^6^ Department of Clinical Medicine School of Basic Medicine Kunming Medical University Kunming Yunnan P. R. China

**Keywords:** immunogenicity, inflammation, lipid nanoparticles, nanotherapy, nanovaccine, nucleic acids

## Abstract

Lipid nanoparticles (LNPs) are transformative vectors for nucleic acid delivery, proven safe and effective in COVID‐19 mRNA vaccines, and enabling advances in cancer immunotherapy and gene editing. However, their inherent immunogenicity presents a double‐edged sword: beneficial adjuvant effects in vaccination can become detrimental inflammatory responses in applications like treating inflammatory/fibrotic diseases or gene editing‐based therapies. This review comprehensively evaluates LNP‐associated inflammation and mitigation strategies. We begin with an in‐depth analysis of molecular mechanisms, detailing how specific lipid components, endocytic pathway activation, and nucleic acid sensing drive immune stimulation. Key modulatory factors, including LNP structural characteristics, administration routes, and biodistribution, are examined. We then explore cutting‐edge engineering approaches to circumvent immunogenicity, encompassing structure‐guided design of ionizable lipids, sophisticated biomimetic strategies using natural membrane coatings, innovative co‐delivery systems incorporating anti‐inflammatory agents, and emerging technologies for immune‐evasive LNPs. By elucidating the intricate relationship between nanocarrier physicochemical properties and host immune recognition, while addressing translational hurdles, this review provides critical insights for developing safer, next‐generation LNPs tailored for diverse therapeutic applications.

## Graphical abstract

Lipid nanoparticles (LNPs) enable efficient nucleic acid delivery, but their immunogenicity is a double‐edged sword. This review explores LNP‐driven innate and adaptive immunity, covering lipid components, endosomal escape, and nucleic acid sensing. It discusses engineering strategies such as biomimetic coatings, anti‐inflammatory co‐delivery, and advanced lipid design, while highlighting key manufacturing and regulatory hurdles to guide safer LNP‐based therapeutics.

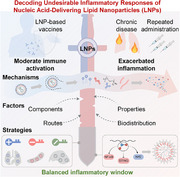

## 1 Introduction

Lipid nanoparticles (LNPs), typically formulated with ionizable lipids, helper lipids, cholesterol, and PEGylated lipids, have revolutionized nucleic acid delivery, as evidenced by the remarkable clinical success of COVID‐19 mRNA vaccines [1, 2]. The ionizable lipid‐mediated endosomal escape mechanism enables efficient cytosolic delivery of different nucleic acid payloads [3, 4]. Notably, previously developed mRNA‐LNP COVID‐19 vaccines achieved protective efficacy rates exceeding 90%, representing the first large‐scale clinical validation of the non‐viral vector technology [5, 6]. This breakthrough rapidly expanded LNP applications across therapeutic areas, evidenced by marketed mRNA vaccines (e.g., Comirnaty and Spikevax) and siRNA therapies (e.g., Onpattro for transthyretin amyloidosis). In November 2023, Japan approved ARCT‐154, a novel saRNA‐LNP vaccine for COVID‐19 prevention [7, 8]. Furthermore, LNP‐based cancer vaccines (like BNT122 and mRNA‐4157) and CRISPR‐Cas9 gene editing therapies (e.g., NTLA‐2001) have advanced to phase 3 clinical trials, though full regulatory approval is pending [9, 10, 11, 12, 13, 14, 15].

However, as LNPs advance toward broader use, the inherent immunogenicity and the resulting inflammatory responses present a distinct double‐edged nature, with clinical impact highly context‐dependent [16, 17]. Specifically, LNP‐triggered immunity provides beneficial adjuvant effects that enhance vaccine immunogenicity, while excessive or uncontrolled inflammation poses significant safety risks. In vaccines, LNP components and nucleic acid payloads can activate innate immune pathways, inducing the production of type I interferons and other cytokines. This promotes the maturation and activation of antigen‐presenting cells (APCs) [18, 19, 20] while concurrently enhancing T‐cell and B‐cell responses, thereby significantly improving vaccine efficacy (e.g., neutralizing antibody titers) [4, 21]. This intrinsic “self‐adjuvant” property makes LNPs efficient nucleic acid delivery platforms. Unfortunately, when immune activation exceeds the optimal threshold, it transitions from beneficial to harmful in non‐vaccine therapeutic scenarios. Excessive or persistent inflammatory responses can trigger adverse reactions ranging from local symptoms (such as redness and pain at the injection site) to systemic manifestations (e.g., fever, fatigue), and in severe cases (e.g., high‐dose intravenous administration), cytokine release syndrome or complement activation‐related pseudo‐allergy (CARPA) [22, 23]. Clinical data indicate that approximately 10%–15% of vaccine recipients experience moderate to severe fever reactions. This dose‐dependent inflammatory toxicity poses significant challenges for repeat‐dosing regimens required in chronic diseases and oncology [24, 25]. Critically, LNP‐triggered inflammation may exacerbate underlying pathology in chronic inflammatory or fibrotic conditions [16, 17, 26]. During in vivo CRISPR‐Cas9‐based gene editing, nucleic acid‐induced innate immune responses can compromise editing efficiency and introduce safety risks [27, 28, 29]. Furthermore, for diseases necessitating long‐term treatment (e.g., cancers, genetic disorders), cumulative inflammatory toxicity narrows the therapeutic window [24, 25, 30]. Collectively, these adverse effects constitute a major translational barrier for broader LNP therapeutic applications.

Given this context‐dependent duality, management strategies for LNP‐induced inflammation should be application‐specific. For vaccines, transient inflammation is fundamentally beneficial, serving as a self‐adjuvanting mechanism to enhance immune responses. Conversely, in non‐vaccine applications requiring chronic or repeated administration, uncontrolled inflammation poses safety concerns demanding mitigation. This review focuses on the latter scenario, exploring mechanisms underlying LNP‐driven inflammatory adverse effects and strategies to control them, while acknowledging that vaccine applications operate under distinct design paradigms. Therefore, a core challenge lies in deciphering the fundamental logic underlying the inflammatory duality of nucleic acid‐delivering LNPs and achieving precise immunogenicity control across therapeutic contexts. Although existing studies have significantly advanced LNP delivery efficiency in various tissues and cell types [29, 31, 32, 33, 34], comprehensive analyses of LNP‐induced inflammatory responses remain scarce. Emerging evidence indicates that inflammatory activation involves complex interactions among multiple factors, such as membrane‐disruptive properties of cationic lipids, Toll‐like receptor (TLR)‐activating nucleic acid motifs, and complement system activation by PEGylated components [35, 36, 37]. The critical challenge is to balance the essential transfection activity of LNPs against maximal suppression of adverse inflammatory responses, thereby broadening their therapeutic application in inflammation‐associated chronic diseases. This review systematically analyzes inflammatory mechanisms and critical determinants from the perspective of the composition and physicochemical properties of LNP, incorporating multidimensional mitigation strategies such as rational design of biodegradable lipids, structure‐guided optimization of nucleic acid components, and anti‐inflammatory agent co‐delivery systems. By elucidating these structure‐immunogenicity correlations, we aim to establish a design framework for developing safer, next‐generation LNPs with controlled immunogenicity profiles, thereby accelerating the clinical translation of precision nanomedicines for genetic therapies while addressing current safety limitations.

## 2 Core Mechanisms of LNP‐Induced Inflammatory Responses

### 2.1 From Inflammatory Responses to Clinical Manifestations

Although the inflammatory properties of LNPs are essential for mRNA vaccine efficacy, excessive or dysregulated inflammation can, under certain conditions, trigger clinical adverse events. Recent studies have provided direct mechanistic links between LNP‐driven inflammation to specific adverse reactions [38, 39, 40, 41]. Systemic reactogenicity, such as fever and fatigue, primarily stems from acute inflammation induced by the LNP itself. Honda et al. demonstrated that the LNP component, rather than the encapsulated mRNA, drives these systemic reactions in mice [38]. Mechanistically, LNP injection rapidly induces interleukin (IL)‐1α, IL‐6, and tumor necrosis factor (TNF)‐α. These cytokines act on cerebral vascular endothelial cells, triggering cyclooxygenase‐2 expression and prostaglandin E2 production, ultimately causing fever. Notably, IL‐6 was identified as a key effector: its neutralization significantly alleviated symptoms like fever and weight loss without compromising the antigen‐specific immune response [38].

In contrast, rare but severe myocarditis following COVID‐19 mRNA vaccination involves a distinct mechanism. This complication results from a T‐cell‐mediated inflammatory cascade initiated by the vaccine [39]. Notably, this process exhibits a “booster effect,” the inflammatory response is markedly amplified after the second dose, mirroring the increased clinical incidence of myocarditis following secondary vaccination [39]. Li et al. further elucidated the upstream mechanisms driving this T‐cell response, demonstrating that the BNT162b2 vaccine induces type I interferons via the MDA5/IFNAR signaling axis. This induction promotes the expansion of antigen‐specific CD8^+^ T cells, potentially contributing to the underlying cellular basis of T‐cell‐mediated inflammatory pathology [40]. Overall, these findings indicate that under certain conditions, the initial inflammatory signals triggered by LNPs can be amplified through adaptive immune memory mechanisms. This application can ultimately drive pathological T‐cell responses, manifesting as rare but severe adverse cardiac events.

Beyond their pathological effects, LNPs play a fundamental role in orchestrating protective adaptive immunity. One study demonstrated that LNPs induce dendritic cells to upregulate CD25 and Ebi2 expression, promoting their migration to the T‐B cell border of lymphoid organs, providing critical signals for follicular helper T (Tfh) cell differentiation and efficient germinal center responses [41]. This dual immunomodulatory role not only promotes protective humoral immunity but also, in rare instances, triggers pathological T‐cell responses, highlighting the complexity of LNP biology. Having established the mechanistic links between LNP‐induced inflammation and clinical outcomes, we next examine the individual contributions of each LNP component to these processes.

### 2.2 Regulation of Inflammation by LNP Components

**FIGURE 1:**
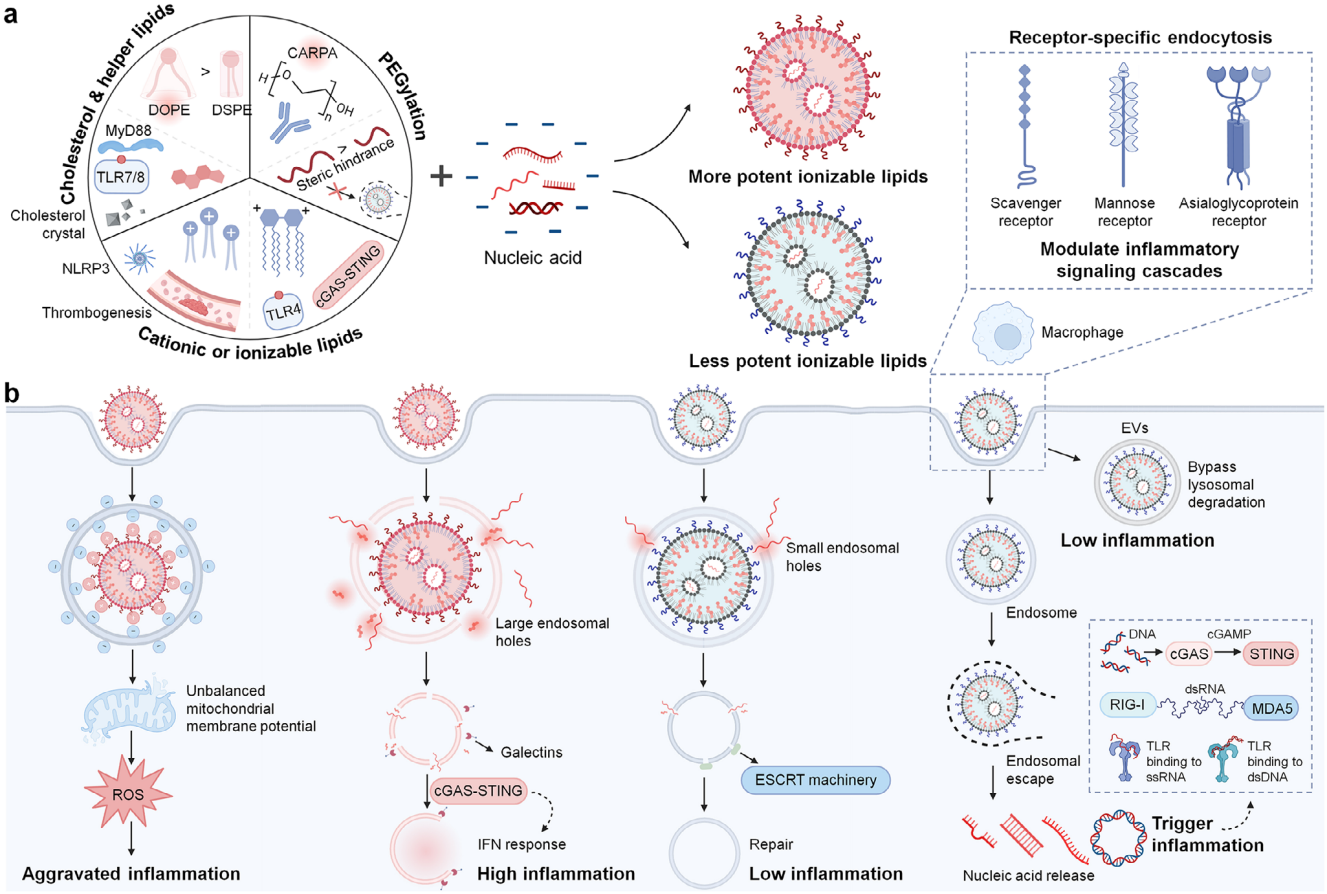
Core mechanisms underlying LNP‐induced inflammatory responses. (a) Inflammation induced by LNP components. Cationic or ionized lipids: Their molecular structure‐dependent membrane instability is critical for endosomal escape, but this property may also directly destabilize endosomal membrane stability, activate PRRs such as TLRs, and initiate proinflammatory signaling pathways. Cholesterol: Excess cholesterol and its derivatives in LNPs increase membrane rigidity, enabling cellular recognition as a “danger signal” that triggers NLRP3 inflammasome assembly and activation. PEGylated lipids: They trigger hypersensitivity reactions and accelerate blood clearance by activating the complement system and inducing the production of anti‐PEG antibodies. Helper lipids: They indirectly affect the intensity of the above processes by regulating the fluidity and stability of the LNP membrane. (b) Amplification of inflammatory signals in internalizing mechanisms. Membrane damage and mitochondrial stress: Unbalanced perturbation of the endocytic membrane can lead to an imbalance of mitochondrial membrane potential and the generation of ROS, which in turn activate inflammatory pathways. Pores formed by membrane damage: Repair of small pores (< 10 nm) is usually mediated by the ESCRT complex and causes only low‐level inflammation. Irreparable macropores (> 50 nm) lead to leakage of cell contents, which act as DAMPs and trigger a high‐intensity inflammatory response. Endocytic pathways: LNPs endocytosis through non‐classical pathways, such as extracellular vesicles, can effectively bypass lysosomal degradation and achieve low‐inflammatory nucleic acid delivery. The classical lysosomal pathway is accompanied by the release of pro‐inflammatory signals during degradation. Immunogenicity of nucleic acid loads: Nucleic acids, such as mRNA, that are successfully released into the cytoplasm can be recognized by cytoplasmic PRRs, constituting another level of immune activation.

The individual components of LNPs modulate inflammatory responses through distinct molecular mechanisms. Cationic/ionizable lipids critically influence endosomal escape efficiency through their molecular structure‐dependent membrane destabilization capacity. Cholesterol and its derivatives can promote inflammasome activation when present in excess stoichiometric ratios. Meanwhile, PEGylated lipids induce hypersensitivity reactions through complement activation and the generation of anti‐PEG antibodies (Table 1 and Figure 1a).

**TABLE 1:** Inflammation induced by common components of nucleic acid‐containing LNPs.

Lipids		Applications	Major inflammatory pathways	Typical symptoms /adverse reactions	References
Ionizable lipids	SM‐102	Spikevax	Activate TLR4 signaling	Redness, swelling, pain, low fever, and neutrophil infiltration at the injection site	[6, 35, 37, 224, 225]
	ALC‐0315	Comirnaty	Complement activation and potent innate immune activation by RIG‐I‐like receptors	Elevated liver enzymes (ALT and bile acids)	[5, 169, 224, 225, 226]
	DLin‐MC3‐DMA	Onpattro	Activate the NLRP3 inflammasome pathway	Muscle pain, complement‐mediated chills, and hypotension	[9, 125, 225, 227, 228]
	C12‐200	Preclinical mRNA delivery	Activate TLR4 signaling	Local neutrophil infiltration	[17, 61]
Helper lipids	DSPC	Spikevax, Comirnaty, and Onpattro	Weak inflammatory responses	Mild local reaction and high stability	[29, 229]
	DOPE	Pre‐clinical formulations	Activate TLR4 signaling	Elicit potent T cell responses	[35, 60, 61, 230, 231, 232, 233, 234]
	DOPS	Pre‐clinical formulations	Elicit CD8^+^ T cell responses	Local neutrophil and macrophage infiltration	[235]
	DOPG	Pre‐clinical formulations	Inhibit macrophages	Attenuated inflammation	[236, 237]
Cholesterol and its analogues	Cholesterol	All LNPs	Oxidation forms oxysterols, which activate TLR4	Foam cell formation, a critical contributor to atherosclerosis	[57, 238]
	β‐Sitosterol	Therapies for cancer and arthritis	Inhibit cholesterol oxidation	Reduced inflammation	[231, 239, 240]
	Oxysterol	Therapies for atherosclerosis	Activate macrophages	Increased oxidative stress and neutrophil infiltration	[147, 238, 241, 242]
	7α‐Hydroxycholesterol	T cell‐targeted therapies	Activate PRRs	Direct cellular damage effect	[241, 243]
PEG lipids	DMG‐PEG_2000_	Spikevax and Onpattro	Anti‐PEG antibody reaction	Allergic reactions and accelerated blood clearance	[62, 244, 245, 246]
	ALC‐0159	Comirnaty	Anti‐PEG antibody reaction	Lower immunogenicity	[62, 68, 244]
	DSPE‐PEG_2000_	Long‐lasting circulation systems	Anti‐PEG antibody reaction	Allergic reactions and accelerated blood clearance	[68, 244, 247]

#### 2.2.1 Cationic/Ionizable Lipids

The immunological activities of LNPs are fundamentally determined by their structural components [36]. While cationic lipids significantly enhance cellular uptake and endolysosomal escape efficiency of LNPs through interactions with negatively charged membranes, their persistent positive charge promotes undesirable plasma protein adsorption, especially fibrinogen binding, which can initiate platelet activation and thrombogenesis [42]. At the molecular level, these cationic components have been shown to activate the NLRP3 inflammasome pathway, resulting in elevated secretion of pro‐inflammatory cytokines, including IL‐1β [43, 44, 45]. To address this challenge, current research has shifted toward developing smart, pH‐responsive ionizable lipids that maintain neutral charge under physiological conditions while selectively acquiring positive charge in acidic endosomal environments. This strategic approach aims to optimize both delivery efficiency and immunological safety profiles [42, 46].

The pH‐responsive properties of ionizable lipids are central to LNP delivery efficiency, with their chemical structures directly determining endosomal membrane destabilization during escape processes. Rapidly metabolizable ionizable lipids (e.g., ester‐containing structures) create transient, nanoscale membrane pores that are efficiently repaired by the endosomal sorting complex required for transport (ESCRT) machinery, thereby minimizing endosomal stress‐related activation of interferon signaling [47]. By contrast, metabolically stable lipids, exemplified by SM‐102 employed in COVID‐19 vaccines, cause sustained endolysosomal membrane perturbation, leading to STING pathway activation and consequent chronic inflammatory signaling cascades [47, 48]. Increasing evidence indicates that oxidized phospholipid derivatives generated during lipid degradation can act as damage‐associated molecular patterns (DAMPs), triggering TLR4‐mediated inflammatory signaling. This underscores the importance of synchronizing lipid degradation kinetics and immune clearance rates [49, 50]. In addition, comparative studies reveal that C12‐200 lipid formulations elicit substantially greater inflammatory cytokine production than SM‐102‐based systems, demonstrating enhanced reactogenicity in mRNA LNP applications. This heightened immunostimulation stems from superior endosomal escape efficiency, increased immune cell uptake, and molecular structure‐dependent innate immune activation of C12‐200. In contrast, SM‐102 achieves an optimal balance between mRNA delivery efficiency and inflammatory tolerance by carefully engineered ionization properties and controlled degradation kinetics, making it more suitable for therapeutic applications requiring repeated or long‐term administration [51]. These findings suggest a clear structure–activity relationship between ionizable lipid chemistry and pro‐inflammatory potential. However, future systematic studies need to incorporate more key lipids (such as ALC‐0315 and MC3) into this comparison framework to comprehensively delineate relationships between ionizable lipid chemical space and their immune properties.

While the structure–activity relationships discussed, particularly the contrasting behaviors of rapidly degradable versus metabolically stable lipids, and the distinct profiles of C12‐200 and SM‐102, provide valuable guidance for LNP design, it is important to recognize that these studies typically examine isolated lipids or discrete parameters in a simplified system. In vivo, however, the immune system perceives LNPs as integrated entities shaped by the interplay of multiple physicochemical properties, dynamic protein corona formation, and spatiotemporal evolution of lipid composition during systemic circulation. For instance, although C12‐200 exhibits superior endosomal escape compared to SM‐102, this advantage correlates with heightened inflammatory cytokine production, an outcome reflecting the integrated effects of cellular uptake, endosomal processing, and innate immune sensing rather than any single parameter. A more comprehensive understanding requires systematic head‐to‐head evaluations across expanded panels of ionizable lipids (including ALC‐0315, MC3, and next‐generation biodegradable lipids) using multi‐parametric readouts that concurrently capture delivery efficiency and immunogenicity. Such comparative frameworks are essential for establishing definitive structure–immunogenicity relationships to enable rational design beyond empirical optimization.

#### 2.2.2 Cholesterol and Helper Lipids

Cholesterol and phosphatidylcholine‐based helper lipids can regulate inflammatory responses by modulating the membrane fluidity of LNPs. An elevated cholesterol content enhances membrane stability but may prolong endolysosomal retention by reducing lysosomal escape post‐cellular internalization, thereby facilitating TLR7/8 recognition of nucleic acid cargo and subsequent MyD88 pathway activation [52, 53, 54]. Beyond structural stabilization, cholesterol modulates endolysosomal membrane fusion efficiency by altering cholesterol distribution within the related compartments. Cholesterol crystal accumulation may activate the NLRP3 inflammasome by destroying the phagolysosome membrane and promoting the release of inflammatory factors such as IL‐1β. Disorders of cholesterol metabolism, including intracellular accumulation, may lead to increased secretion of inflammatory factors by regulating the immune response of myeloid cells such as macrophages [44]. In contrast, cholesterol derivatives such as cholesteryl hemisuccinate enhance endolysosomal escape, reducing intracellular nucleic acid retention and mitigating sustained TLR3/7/9 activation [55, 56, 57, 58]. Beyond synthetic derivatives, substituting cholesterol with naturally occurring plant sterols offers an alternative strategy to modulate reactogenicity. Replacing cholesterol with stigmastanol or β‐sitosterol maintained antigen‐specific antibody and CD8^+^ T cell responses comparable to control formulations, while significantly reducing inflammatory cytokine production (including IL‐6, TNF‐α, and type I interferons) and thus alleviating fever [59]. Notably, the anti‐inflammatory effect depended on the alkyl chain length at the C24 position of the sterol, suggesting that subtle structural variations can differentially modulate inflammatory outcomes [59]. Collectively, these findings highlight the biphasic and structure‐dependent effects of cholesterol and its derivatives. This dual nature underscores the need to both precisely regulate the cholesterol content (typically optimized at 30 to 40 mol%) and develop functionalized cholesterol derivatives that target membrane behavior modulation.

Different phosphatidylcholine‐based helper lipids cause varied degrees of inflammation due to their spatial structures. Cone‐shaped DOPE promotes membrane fusion and improves cellular uptake and transfection efficiency compared to columnar DSPC. However, it also enhanced the recognition of pathogen‐associated molecular patterns, leading to stronger TLR pathway activation [60]. Thus, the efficient delivery capacity of C12‐200 acts in concert with DOPE perturbation at the cell membrane, leading to greater RNA sensing and amplification of inflammatory signals [61]. In addition to structural factors, the saturation status of phospholipid acyl chains critically governs inflammatory outcomes. Systematic evaluation revealed that replacing saturated DSPC with unsaturated phospholipids (e.g., DOPC or DOPE) maintained robust humoral and cellular immune responses while significantly reducing fever and lowering plasma levels of IL‐6, TNF‐α, and type I interferons [59]. This reductiom was mechanistically linked to the oleic acid tails of unsaturated phospholipids, which suppress pro‐inflammatory signaling pathways more effectively than stearic acid released from DSPC degradation [59]. These findings collectively highlight the necessity to balance delivery efficiency and membrane perturbation when selecting helper lipids, while also demonstrating that judicious selection of phospholipid acyl chain saturation can further decouple immunogenicity from reactogenicity.

#### 2.2.3 PEGylation

PEGylation reduces protein adsorption and mononuclear phagocyte system clearance, yet prolonged use of PEG derivatives induces anti‐PEG antibody production, which may trigger CARPA [23, 62, 63]. Available studies indicate an inverted U‐shaped correlation between the PEG chain length and anti‐inflammatory efficacy. Specifically, lipid derivatives with short‐chain PEGs, such as DMG‐PEG_2000_, maintain colloidal stability of LNPs without fully blocking their target cell uptake, whereas long‐chain PEGs enhance stealth properties but impair endolysosomal escape via steric hindrance, thus increasing nucleic acid leakage risks [64, 65, 66]. Systematic evaluation further demonstrated that shortening the PEG chain length (e.g., PEG_1000_) or reducing the PEG‐lipid molar ratio (e.g., 0%) enhanced antigen‐specific CD8^+^ T cell responses. Conversely, excessive PEGylation (≥2.5% molar ratio or PEG_5000_) diminished both humoral and cellular immunity [59]. Notably, formulations with lower PEG contents exhibited elevated inflammatory cytokine production, while those with higher PEG contents showed reduced reactogenicity, underscoring the delicate balance required between immunogenicity and adverse reactions [59]. In this context, dynamic PEGylation strategies, such as acid‐labile linkers, have emerged as advanced approaches to dissociate PEG shields during late‐stage delivery, thereby harmonizing immune evasion and payload release efficiency [67, 68].

### 2.3 Amplification of Inflammatory Signals in Internalizing Mechanisms

During cellular internalization of LNPs, ionizable lipids induce inflammatory signaling by activating pattern‐recognition receptors (PRRs) (e.g., TLR4). The ensuing endosomal membrane disruption triggers a ROS burst and consequent oxidative stress, thereby amplifying the inflammatory cascade. Notably, the substantial membrane destabilization accompanying endosomal escape elicits dual pro‐inflammatory mechanisms: inflammasome activation via galectin‐mediated danger signal recognition and engagement of cytoplasmic nucleic acid‐sensing pathways through liberated endogenous nucleic acids. These synergistic events culminate in robust amplification of downstream inflammatory signaling networks (Figure 1b).

#### 2.3.1 Inflammatory Signals in Endocytic Mechanisms

The endocytic mechanisms of LNPs significantly depend on cell types and surface modifications. Macrophages predominantly internalize LNPs via scavenger receptor and mannose receptor‐mediated pathways, with galactose‐modified LNPs demonstrating 2.2‐fold higher transfection efficiency than Lipofectamine 6000 through asialoglycoprotein receptor‐mediated uptake [69]. This receptor‐specific endocytosis may modulate inflammatory signaling cascades, as asialoglycoprotein receptor binding drives macrophage polarization toward the M2 phenotype, accompanied by CD98 downregulation and cytokine profile remodeling characterized by reduced TNF‐α and IL‐6 production alongside elevated IL‐10 secretion [69]. However, inflammatory microenvironments dynamically regulate LNP processing. For example, TLR4 activation, while not altering LNP uptake quantities, suppresses mRNA translation via activating downstream protein kinase R (PKR) [70], suggesting that inflammatory signals may compromise LNP efficacy through translational blockade. Structural disparities between LNPs and traditional lipoplexes, in particular charge distribution and supramolecular organization, dictate different endocytic routes. The co‐assembled siRNA‐lipid architecture in LNPs facilitates efficient cellular entry, whereas lipoplexes exhibit membrane surface retention of siRNA, resulting in lower endocytic efficiency [71]. Notably, LNP internalization processes may facilitate lateral propagation of inflammatory signals through extracellular vesicle (EV)‐mediated pathways. Partial internalized LNPs package mRNA into EVs [72], which bypass lysosomal degradation during systemic delivery. However, EV‐associated cargo elicits lower inflammatory cytokine levels compared to parent LNPs, indicating that LNP‐induced inflammation is regulated not only by direct cellular uptake but also via paracrine signaling [72, 73]. These findings emphasize the critical interplay between LNP physicochemical properties (e.g., surface ligands, charge characteristics) and inflammatory microenvironment dynamics in determining endocytic pathway selection and subsequent immunomodulatory outcomes.

#### 2.3.2 Aggravated Inflammation by ROS Generation and Oxidative Stress

Although there is increasing evidence that LNPs have the potential to amplify oxidative stress through inflammatory signaling cascades [74, 75], the current understanding of LNP‐mediated ROS generation is still in its infancy. For instance, when LNPs were used to deliver proangiogenic vascular endothelial growth factor A (VEGF‐A) mRNA, upregulation of proinflammatory cytokines (like IL‐6) was detected in secreted EVs [73]. As well recognized, an inflammatory environment typically accompanies elevated ROS levels [74]. In addition to inhibiting mRNA translation through PKR phosphorylation [70], TLR4 activation may also stimulate ROS production simultaneously by activating the NF‐κB pathway, though the mechanistic link needs to be experimentally verified [75]. The physicochemical properties of LNPs, such as surface charge, lipid composition, and metabolic kinetics, play an important role in regulating intracellular ROS generation [76]. Cationic lipids such as YSK13‐C3 strongly interact with the negative charge of the cell membrane, which can trigger an imbalance of the mitochondrial membrane potential and lead to a burst of ROS [44, 77]. Excessive accumulation of ROS further activates the NLRP3 inflammasome pathway and promotes the secretion of proinflammatory factors such as IL‐1β and IL‐18 [78]. More recently, biodegradability has been demonstrated to be a key determinant of LNP‐induced ROS generation. Rapidly degradable ionizable lipids compatible with ESCRT‐mediated membrane repair systems induce low ROS accumulation, whereas non‐degradable long‐chain lipids, commonly used in clinical formulations, sustain oxidative stress [44, 47]. Despite these advances, the causal relationship between LNPs and ROS regulation remains inadequately characterized. Consequently, direct experimental studies on LNP‐induced ROS dynamic changes are warranted. Advanced models are particularly well‐suited to address this need. For instance, organ‐on‐a‐chip platforms (e.g., liver‐on‐a‐chip) permit real‐time ROS monitoring in physiologically relevant microenvironments. Specific knockout mouse models (e.g., Nlrp3^−/−^, Tlr4^−/−^, Nox2^−/−^, or Nrf2^−/−^) enable targeted dissection of causal signaling pathways. Leveraging these complementary approaches will elucidate the causal relationships between LNP physicochemical properties, ROS production, and downstream inflammatory cascades.

#### 2.3.3 Immune Activation via Endosomal Escape

The release of nucleic acids from LNPs via endosomal membrane disruption is fundamentally regulated by dynamic interactions between lipid components and endosomal membranes. Endosomal escape efficiency relies on the pH sensitivity of ionizable lipids, which undergo protonation in acidic endosomal environments, destabilizing membrane structures to form nanopores for nucleic acid release [47]. In addition, the biodegradation rate of ionizable lipids directly modulates the extent of endosomal membrane damage. Rapidly degradable lipids (such as the ESCRT‐compatible type lipids) generate smaller endolysosomal pores (< 10 nm), enabling mRNA/siRNA release while minimizing lysosomal leakage through membrane repair mechanisms, thereby reducing inflammatory risks. In contrast, non‐degradable conventional lipids form larger pores (>50 nm), causing lysosomal enzyme leakage and activating the cGAS‐STING pathway, which perpetuates interferon (IFN) responses [47, 79]. These findings provide a mechanistic foundation for developing LNPs capable of balancing payload delivery and biocompatibility [78].

Furthermore, endosomal membrane rupture not only facilitates nucleic acid leakage but also increases lysosomal membrane permeability, induces mitochondrial damage, releases ROS and cytochrome C, and activates the caspase‐dependent apoptosis pathway. Apoptotic bodies further release DAMPs, such as high mobility group box 1 and ATP, which amplify NLRP3 inflammasome activation via TLR4 or P2X7 receptors, establishing a pro‐inflammatory positive feedback loop [78, 79, 80, 81]. These interconnected mechanisms highlight the dual role of LNPs as both delivery vehicles and inflammatory mediators, making it necessary to comprehensively optimize lipid chemistry and nucleic acid purity to achieve therapeutic efficacy with minimized immunotoxicity.

### 2.4 Immune Activation via Nucleic Acid Release

Nucleic acids released post‐endosomal escape serve not only as therapeutic agents but also as potent inflammatory triggers. Distinct categories of nucleic acid molecules show significant heterogeneity in eliciting host immune responses and inflammatory processes, with their mechanistic actions intricately associated with structural characteristics, PRRs, and delivery systems [82, 83, 84, 85, 86]. Double‐stranded DNA (dsDNA) activates the cytosolic cGAS‐STING pathway to induce robust type I IFN responses and production of pro‐inflammatory cytokines, like IL‐6 and TNF‐α. While this immunostimulatory property has been exploited in vaccine adjuvant development, hyperactivation may predispose to autoimmune disorders [85, 86]. Plasmid DNA (pDNA), as a circular dsDNA structure, similarly engages this pathway to activate innate immunity, though its inflammatory potential can be modulated through vector design and administration strategies [87, 88].

In contrast, single‐stranded antisense oligonucleotides and small interfering RNAs (siRNAs) exhibit relatively low immunogenicity. Nevertheless, unmodified forms remain capable of activating endosomal TLR7/8 receptors, triggering NF‐κB signaling cascades and subsequent cytokine release [89, 90]. Chemical modifications such as 2′‐O‐methylation and locked nucleic acids substantially reduce immune recognition while enhancing target‐specific gene silencing efficacy [91]. Messenger RNA (mRNA) displays dual immunomodulatory characteristics. Unmodified mRNA triggers potent type I IFN responses [92]. However, nucleoside modifications, such as pseudouridine incorporation, attenuate this response while maintaining translational capacity, a critical feature successfully implemented in COVID‐19 vaccine design [93]. Single‐stranded RNA can activate myeloid cells such as dendritic cells and macrophages through TLR7/8 and induce the release of cytokines (such as IFN‐α/β and IL‐6), while double‐stranded RNA (dsRNA) impurities exacerbate inflammation by the RIG‐I/MDA5 pathway [94, 95]. MicroRNAs (miRNAs) indirectly modulate inflammatory cascades through regulation of immune‐related gene expression, with miRNA mimics demonstrating precise functional regulation of immunocytes via exosome‐based delivery platforms [96, 97]. In summary, LNPs and their nucleic acid components activate innate immunity through a variety of PRRs. To enhance clarity, we systematically elucidate the recognition mechanisms of distinct TLRs and their regulatory strategies in Table 2.

**TABLE 2:** Activation of TLRs by nucleic acid/LNP components and inflammatory regulation strategies.

TLRs	Cellular localization	Stimulating components	Potential mitigation strategies	References
TLR3	Endosomal membrane	dsRNA contaminants produced by in vitro transcription of mRNA, or double‐stranded RNA structures formed by self‐reunion	Optimize the mRNA production process and purification process (such as HPLC purification) to completely remove dsRNA impurities Design sequences to reduce secondary structures	[197, 248, 249]
TLR4	Cell membrane surface	Certain ionizable lipids (such as SM‐102) or their degradation products (such as oxidized phospholipids)	Design biodegradable lipids that degrade rapidly (such as lipids with ester bonds) Use TLR4 antagonists or screen a low immunogenicity lipid library	[35, 250, 251, 252]
TLR7/8	Endosomal membrane	Single‐stranded RNA, especially guanosine‐ and uridine‐rich sequences	Replace uridine with modified nucleosides Optimize sequences (reduce the uridine content and avoid specific motifs) Co‐deliver small molecule inhibitors targeting TLR7/8	[94, 253, 254, 255]
TLR9	Endosomal membrane	Unmethylated CpG sequences	Use CpG motifs (methylation or point mutations)	[256, 257]

The immunogenicity of LNPs may be modulated by their nucleic acid release kinetics, though the underlying mechanism and causal relationship require further experimental elucidation. Following endocytosis, LNPs release their nucleic acid cargo into the cytoplasm. For mRNA, this release process is sufficient, but for DNA, there are additional diffusion barriers. Inefficient release delays nucleic acid accumulation in endosomes/lysosomes, which may trigger unwanted inflammatory responses [65], since lysosomal accumulation activates inflammatory pathways via PRR‐mediated detection of nucleic acid fragments, potentially inducing cytokine storm [98]. Notably, the currently reported endosomal escape efficiency of LNPs is generally low, usually less than 2.5% [99], which further increases the possibility of nucleic acid retention in endosomes, prolongs its interactions with receptors such as TLRs, and continuously activates the innate immune response [99, 100, 101, 102]. In addition, the sustained release process of nucleic acids may exacerbate the instability of nucleic acids in the degradation environment, and their fragments may act as stronger immune stimulation signals to amplify the inflammatory response [103]. In terms of regulatory strategies, some studies have shown that siRNA release from LNPs is significantly affected by environmental factors, such as pulmonary surfactant or heparin, which can accelerate nucleic acid release by disrupting the LNP structure [104]. This suggests that rationally engineered LNPs or exploitation of tissue‐specific microenvironments can achieve targeted cytoplasmic release, minimizing off‐target exposure (e.g., to circulating immune cells) and potentially reducing systemic immune activation risks [99, 101, 105]. To directly investigate the relationship between release kinetics and inflammatory outcomes, several real‐time measurement techniques have been developed. These include fluorescence resonance energy transfer‐based imaging, fluorescence co‐localization analysis, pH‐sensitive probes, and single‐particle tracking, enabling quantitative correlations between release efficiency, subcellular trafficking, and downstream immune activation pathways.

Despite the availability of such tools, most existing mechanistic studies indirectly support the hypothesis that efficient release reduces immunogenicity [100, 106, 107], direct experimental validation remains lacking, particularly quantitative profiling of in vivo inflammatory biomarkers and kinetic correlations between release dynamics and inflammation. Moreover, the reported low endosomal escape efficiency (< 2.5%) raises a fundamental unresolved question: Do endosomally trapped LNPs disproportionately drive inflammatory signaling, or is inflammation primarily driven by the minor fraction achieving successful escape? Resolving this dichotomy is critical for evaluating whether improving escape efficiency represents a viable strategy for reducing LNP reactogenicity. Furthermore, LNP‐induced inflammation arises from the combined effects of lipid components and nucleic acid cargo. However, the relative contributions of these two sources, i.e., lipid‐mediated (e.g., NLRP3 inflammasome activation, STING signaling) versus nucleic acid‐mediated (e.g., TLR7/8, cGAS‐STING) inflammatory signals, remain poorly dissected. Establishing quantitative frameworks to disentangle these contributions and link release kinetics to inflammatory outcomes is essential for rationally designing LNPs that minimize immunogenicity while preserving delivery efficiency.

### 2.5 Adaptive Immune Responses: The Challenge of Repeated Dosing

While the preceding sections have focused on innate immune activation, such as TLR signaling, inflammasome engagement, and ROS generation, these early events also serve as critical gateways for the adaptive immune system. For non‐vaccine LNP applications that require repeated administration (e.g., chronic diseases, gene editing‐based therapies), adaptive immune responses pose a formidable challenge that can undermine therapeutic efficacy and patient safety.

#### 2.5.1 LNP‐Mediated Activation of Adaptive Immunity

Upon administration, LNPs are actively taken up by antigen‐presenting cells (APCs), such as dendritic cells and macrophages, at the injection site and in draining lymph nodes [108]. This uptake serves a dual function: it delivers mRNA‐encoded antigens to APCs, while the ionizable lipid component of LNPs simultaneously acts as a danger signal, inducing the production of pro‐inflammatory cytokines, particularly IL‐1β and IL‐6 [16]. This immunostimulatory microenvironment promotes APC maturation and drives the differentiation of Tfh cells and germinal center B cells, which are essential for generating high‐affinity antibody responses [108]. The particulate properties of LNPs, including the size, surface charge, and lipid composition, further influence their immunogenicity by modulating uptake efficiency, APC activation profiles, and endosomal escape [109]. Upon adaptive immune activation, vaccine‐induced or pre‐existing antibodies against LNP components can mediate several clinically relevant phenomena, including accelerated blood clearance (ABC), CARPA, and T cell‐mediated immune responses [16, 108].

#### 2.5.2 Anti‐PEG Antibodies and ABC Phenomenon

The PEGylation strategy, widely used to prolong circulation time and reduce opsonization, paradoxically becomes a major source of immunogenicity upon repeated dosing. Pre‐existing anti‐PEG antibodies are detectable in approximately 20%–40% of humans due to ubiquitous exposure to PEG‐containing products in consumer goods (e.g., cosmetics, food) and pharmaceuticals. These antibodies can bind to PEGylated LNPs upon first administration, triggering complement activation and rapid clearance [110]. Moreover, even in individuals without pre‐existing antibodies, the initial LNP dose can induce de novo anti‐PEG IgM and IgG responses, primarily through a T cell‐independent mechanism involving marginal zone B cells in the spleen [111]. Upon subsequent dosing, these antibodies opsonize LNPs, leading to ABC, a phenomenon characterized by dramatically reduced circulation time, diminished therapeutic efficacy, and enhanced accumulation in the liver and spleen [112]. This ABC effect has been documented in multiple preclinical studies and has emerged as a critical barrier for LNP‐based therapies requiring chronic or repeated administration.

To mitigate PEG immunogenicity, multiple strategies have been explored. One approach involves replacing PEG with alternative hydrophilic polymers such as polyglycerol, polyoxazoline, or zwitterionic polymers. For example, polyglycerol‐functionalized NPs have demonstrated reduced anti‐polymer antibody responses compared to PEGylated counterparts in repeated‐dose studies [113]. Another promising strategy employs biodegradable PEG‐lipids with cleavable linkers. PEG‐lipids containing an ester bond between the PEG chain and the lipid anchor can undergo hydrolysis by esterases in the circulation, leading to gradual PEG shedding. This design reduces PEG surface exposure over time, potentially minimizing B‐cell activation and subsequent anti‐PEG antibody production while maintaining initial stealth properties. Studies have shown that such cleavable PEG‐lipids significantly reduce the ABC phenomenon in repeated‐dose regimens compared to conventional non‐cleavable PEG‐lipids [112]. Additionally, reducing PEG density or chain length has been shown to modulate immune responses, although the direct impact on anti‐PEG antibody production and ABC requires further evaluation [59]. Given these clinical implications, patient stratification based on pre‐existing anti‐PEG antibody levels may be necessary to identify individuals at elevated risk of ABC or infusion reactions, potentially guiding personalized dose selection or pre‐medication regimens [110].

#### 2.5.3 CARPA and Infusion Reactions

CARPA is a non‐IgE‐mediated hypersensitivity reaction triggered by NP‐based therapeutics, characterized by cardiopulmonary distress, hypotension, and in severe cases, anaphylaxis [114]. Unlike classical allergic reactions, CARPA is driven by direct complement activation via the NP surface, which occurs either through the antibody‐independent alternative pathway or the classical pathway when anti‐PEG antibodies are bound to the particle surface [115]. PEGylated LNPs can activate the complement system through both mechanisms, leading to the generation of anaphylatoxins C3a and C5a, which trigger mast cell and basophil degranulation, thus releasing histamine and other vasoactive mediators [114, 115].

The clinical significance of CARPA is well‐documented across nanomedicines. For the liposomal formulation Doxil (doxorubicin), infusion reactions characterized by cardiopulmonary distress occur in a subset of patients, which have been attributed to complement activation [114]. As for the siRNA‐LNP product Onpattro (Patisiran), infusion‐related reactions are a recognized safety concern, and pre‐infusion of corticosteroids, antihistamines, and acetaminophen is recommended to mitigate these reactions. Notably, the incidence of infusion reactions correlates with anti‐PEG antibody titers, suggesting that anti‐PEG antibodies contribute to complement activation and CARPA [9, 110].

To mitigate the risk of CARPA, different strategies have been explored. Reducing PEG density or using alternative hydrophilic polymers can lower complement activation by minimizing the surface area available for complement factor binding [113, 114]. Surface PEG on liposomes can promote complement activation via negatively charged phospholipid anchors (e.g., DSPE), and modification of these anchor structures can modulate complement activation profiles [115]. Complement inhibitors, such as anti‐C5 antibodies (eculizumab) or C5a receptor antagonists, have shown efficacy in blocking CARPA in preclinical models, though their routine clinical use for LNP infusion remains limited due to cost and safety concerns [114]. Screening for pre‐existing anti‐PEG antibodies has been proposed to identify patients at elevated risk of infusion reactions, enabling personalized prophylactic strategies [110].

#### 2.5.4 T Cell‐Mediated Adaptive Immunity to LNP Components

Beyond humoral responses, LNPs can elicit T cell‐mediated adaptive immunity through the expressed protein antigen. The particulate nature of LNPs may enhance cross‐presentation of encapsulated antigens to antigen‐presenting cells, thereby driving CD8^+^ T cell activation [116]. LNP composition further modulates this response: studies indicate that shortening PEG chain length or reducing the PEG‐lipid molar ratio can increase CD8^+^ T cell activation and inflammatory cytokine production [59]. Such T cell responses pose potential challenges for non‐vaccine applications that require long‐term persistence of target cells.

In CRISPR‐Cas9 gene editing, delivery of Cas9 mRNA or protein can elicit T cell responses. Pre‐existing T cell reactivity to *Streptococcus pyogenes* Cas9 has been detected in 67%–78% of healthy adult donors, with a similar prevalence observed for *Staphylococcus aureus* Cas9 [117]. For mRNA‐based protein replacement therapies, the expressed therapeutic protein may be recognized as foreign, raising concerns about neutralizing antibody responses and potential T cell‐mediated clearance, particularly when the therapeutic protein is absent or non‐functional in the patient, which increases the likelihood of it being recognized as a foreign antigen [118]. The presence of pre‐existing T cell immunity to Cas9 underscores the need for comprehensive immunogenicity assessment during preclinical and clinical development. Patient stratification based on pre‐existing T cell responses may be essential to identify individuals at risk of diminished efficacy or adverse reactions, as has been explored in the context of AAV gene therapy [119]. To enable repeated dosing in chronic applications, strategies such as the use of alternative Cas9 orthologs, transient immunosuppression, or the development of alternative delivery platforms may be necessary.

## 3 Modulating Factors Dominating LNP‐Induced Inflammatory Outcomes

While the core mechanisms described above define how LNPs trigger inflammation, the ultimate intensity, duration, and localization of the inflammatory response are critically influenced by a series of modulating factors, such as physicochemical properties of LNPs, administration routes, and resultant tissue biodistribution profiles.

### 3.1 Structural Properties of LNPs and Their Association With Inflammatory Activation

**FIGURE 2:**
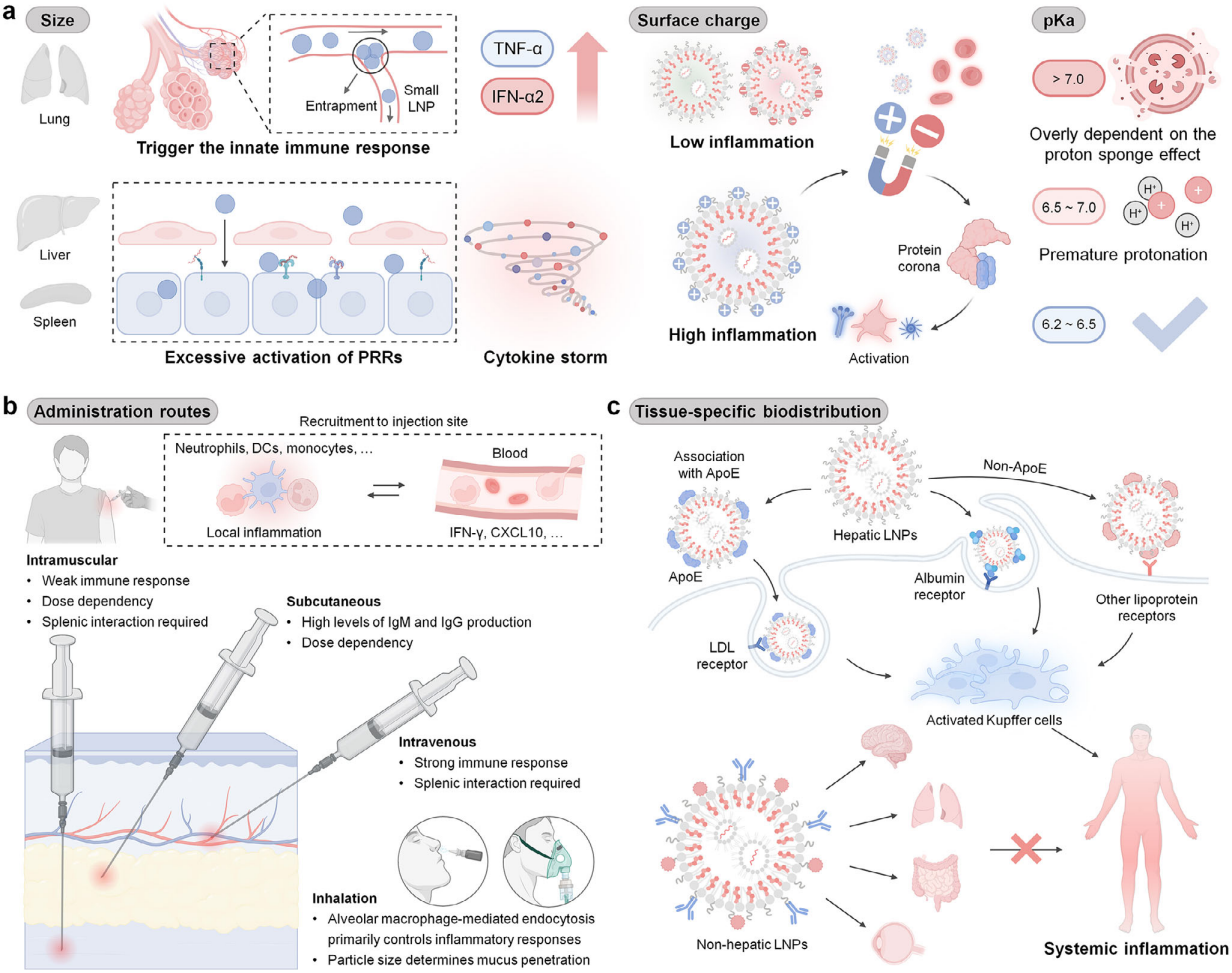
Modulating factors dominating LNP‐induced inflammatory outcomes. (a) Structural properties of LNPs and their association with inflammatory activation. The immunogenicity of LNPs is intrinsically governed by their structural parameters: Particle size dictates immune cell recognition and organ targeting, thereby modulating inflammatory response location and intensity; Surface charge indirectly regulates inflammation through protein corona composition and immune cell uptake efficiency; The pKa of ionizable lipids determines endosomal escape efficiency and membrane perturbation extent, serving as a critical determinant of immunogenicity threshold. (b) Impact of administration routes on inflammatory responses. Delivery routes determine the immune milieu of initial LNP exposure, driving distinct inflammatory outcomes: Intramuscular/subcutaneous injection mainly causes local inflammation, which is often manifested as redness and pain at the injection site, with mild systemic effects; Intravenous injection leads to systemic LNP distribution, which may potentiate robust inflammation (such as fever and cytokine release) and complement‐mediated pseudoallergy risk; Intranasal administration tends to activate mucosal immunity, which is beneficial to defense against respiratory tract infections, with minimal systemic inflammation. (c) Regulation of inflammatory responses by tissue‐specific biodistribution patterns. LNP biodistribution dictates organ‐specific inflammatory responses: ApoE‐mediated liver targeting promotes hepatocyte uptake, establishing the liver as the primary inflammatory site with associated hepatotoxicity (e.g., transaminitis); Extrahepatic targeting (e.g., spleen, lung, brain tissues) through non‐ApoE‐mediated/non‐liver targeting pathways circumvents systemic inflammation but may trigger tissue‐specific immunotoxicities.

Physicochemical properties of LNPs affect their inflammatory responses through multiple interdependent mechanisms. The particle size directly modulates immune recognition efficiency, with optimal dimensions determining cellular uptake patterns and subsequent immune activation. Surface charge characteristics, particularly positive zeta‐potential, significantly influence inflammatory intensity by facilitating complement system activation and TLR pathway engagement through enhanced interactions with immune cell membranes. Concurrently, the pKa values of ionizable lipids govern inflammatory signaling cascades by controlling the kinetics and efficiency of endosomal escape, which in turn affects the exposure of nucleic acid payloads to cytoplasmic sensors. These structure–activity relationships collectively determine the immunogenicity profile of LNPs (Figure 2a), highlighting the delicate balance between delivery efficiency and inflammatory responses inherent to LNP design.

#### 3.1.1 Particle Size

Particle size serves as an important aspect affecting the biodistribution and inflammatory potential of LNPs. LNPs with a smaller particle size (< 150 nm) are more diffused throughout the systemic circulation and more easily enter tissues or organs through the vascular barrier. In organs such as the lungs, small particles are more efficiently internalized by specific cells, including alveolar epithelial cells, alveolar macrophages, and hematopoietic stem cells [120]. However, they are more likely to trigger innate immune responses, thus inducing the release of proinflammatory cytokines (e.g., IFN‐α2 and TNF‐α) [121, 122]. By contrast, larger LNPs (> 200 nm) tend to accumulate in the liver or spleen, where their rapid clearance by the reticuloendothelial system or excessive activation of PRRs may exacerbate the inflammatory response. In this aspect, they are more suitable for local administration, but their prolonged retention at the injection site may trigger local innate immune responses, such as recruitment of monocytes and neutrophils, leading to tissue inflammation [123, 124]. Studies have shown that the size and mRNA encapsulation efficiency of LNPs significantly affect the distribution and early immune response of mRNA vaccines in vivo. Smaller MC3 LNPs (114 nm) migrate more quickly to the lymph nodes and elicit a strong inflammatory response, whereas larger DOG‐IM4 LNPs (184 nm) persist longer and have a weaker inflammatory response at the injection site [125]. Moreover, precise regulation of particle size distribution by microfluidic technology can minimize off‐target immune activation. These findings highlight the need to tailor LNP dimensions to optimize therapeutic efficacy while avoiding unexpected immunostimulatory effects.

#### 3.1.2 Surface Charge

The surface charge of LNPs controls their interactions with cellular membranes and immune components. Cationic LNPs enhance cellular uptake through electrostatic adsorption, yet their high positive surface potentials (> +20 mV) promote binding with negatively charged plasma proteins, forming a “protein corona” that may activate the complement system, platelets, or the NLRP3 inflammasome, ultimately triggering the release of pro‐inflammatory cytokines [126]. In contrast, neutrally charged or mildly anionic LNPs minimize nonspecific plasma protein interactions, thereby reducing inflammatory responses [127]. Recent advances in surface engineering highlight the importance of charge modulation. By neutralizing the negative charge of siRNA with tropomyosin, the developed LLC‐NPs achieved high gene silencing activity and improved safety at a low cationic lipid/siRNA charge ratio [128]. Zwitterionic molecular coatings or PEGylation strategies allow precise control of surface potentials. Similarly, siRNA‐loaded LNPs derived from zwitterionic polycarboxybetaine‐modified lipids (ZP‐LNPs), with near‐neutral surface charge, demonstrate remarkably reduced protein adsorption while mitigating thrombogenicity and inflammatory responses [105]. Notably, whereas surface charge positively correlates with the inflammation intensity, complete neutralization of cationic charge would weaken the electrostatic interaction of LNPs with target cell membranes, potentially reducing targeting efficacy and leading to nonspecific distribution or accelerated blood clearance [129]. These studies demonstrate the critical role of optimizing charge microenvironments to balance cellular uptake efficiency with immune compatibility. By integrating charge‐tuning technologies, next‐generation LNPs may achieve notably enhanced targeted delivery while avoiding systemic immunotoxicity.

#### 3.1.3 pKa

The pKa value of LNP‐forming lipids modulates their ionization state in physiological environments and endosomal escape capacity, directly influencing nucleic acid release efficiency and inflammatory signaling pathways [130]. LNPs based on lipids with pKa values between 6.5 and 7.0 undergo premature protonation in the bloodstream, leading to nonspecific cellular membrane fusion and failed lysosomal escape. This results in endosomal membrane destabilization, elevated ROS production, and subsequent activation of pro‐inflammatory cascades [47, 79]. In contrast, carriers with excessively high pKa are overly dependent on the proton sponge effect, which may induce lysosomal membrane rupture, release of cathepsins, and NLRP3 inflammasome activation. To address this, degradable ionizable lipids have been engineered to balance endosomal escape efficiency with inflammatory risks. These lipids selectively protonate in the acidic endosomal environment (pH 5–6), facilitating nucleic acid release via non‐lamellar phase transitions while minimizing lysosomal membrane disruption and inflammasome activation [131, 132]. Consequently, ionizable lipids are typically designed with pKa values between 6.2 and 6.5, ensuring neutrality in the bloodstream (pH 7.4) to reduce nonspecific cellular interactions and prolong circulation. Upon entering endosomes, protonation confers a positive charge, enhancing interactions with endosomal membranes [133, 134]. A notable example is LNP 55, which achieves high‐efficiency localized delivery in the placenta while mitigating systemic inflammation by optimizing pKa compatibility with the target tissue microenvironment [135]. This strategy highlights the importance of coordinating therapeutic efficacy and immunomodulatory safety through individualized pKa, offering a blueprint for next‐generation LNP designs in precision medicine.

The previous sections have discussed the effects of particle size, surface charge, and pKa value on LNP inflammatory responses. These structure–activity relationships provide valuable guidance for the rational design of LNPs. However, it is worth noting that discussing each parameter in isolation effectively simplifies a complex issue. In a real physiological environment, what the immune system perceives is not any single isolated parameter, but rather the overall state of LNPs under specific spatiotemporal conditions, a process also modulated by microenvironmental factors such as ions and proteins. Taking surface charge as an example: as mentioned earlier, cationic LNPs readily adsorb plasma proteins to form a protein corona. This means that the initial surface charge we measure is quickly covered by the protein corona upon entering the body, and what actually interacts with immune cells is the protein corona rather than the original lipid surface. In other words, the immune system “sees” an LNP that has been remodeled by its microenvironment.

Similarly, while the pKa value influences endosomal escape efficiency, this effect is intertwined with lipid degradation kinetics, particle size, and local pH changes within the microenvironment. Examining pKa alone makes it difficult to fully predict the ultimate intensity of the inflammatory response. In fact, these parameters do not operate independently: particle size influences surface curvature, which in turn affects the composition of the protein corona, and the composition of the protein corona then alters the actual presentation of surface charge; pKa determines the efficiency of endosomal escape, but the extent of membrane perturbation during escape is also associated with particle size and lipid degradation kinetics. These factors are interwoven and collectively dictate the final inflammatory outcome. Therefore, in the design and optimization of LNPs, simply pursuing an optimal value for any single parameter is often insufficient to achieve the desired effect; instead, these factors need to be considered comprehensively as a whole. Future research should shift from static parameter characterization toward dynamic, holistic evaluation, examining how these parameters interact synergistically in models that more closely mimic physiological conditions, rather than optimizing any single indicator in isolation.

### 3.2 Impact of Administration Routes on Inflammatory Responses

Inflammatory responses of LNPs exhibit pronounced route‐dependent characteristics, with each administration method eliciting distinct spatial and temporal patterns of immune activation. Intramuscular injection primarily induces localized neutrophil recruitment as well as mild and transient inflammatory responses, making it particularly suitable for vaccine applications where controlled immunostimulation is desirable. In contrast, intravenous delivery leads to preferential hepatic and splenic accumulation, creating risks of systemic cytokine release syndrome and complement activation‐related pseudoallergy. Pulmonary administration triggers compartmentalized inflammatory cascades in the bronchial–alveolar microenvironment, while intraperitoneal injection provokes acute but self‐limiting peritoneal inflammation accompanied by significant systemic exposure due to the extensive lymphatic drainage and vascularization of the peritoneal cavity. These route‐specific immunological signatures underscore the critical importance of administration pathway selection in balancing therapeutic efficacy against inflammatory side effects during LNP development (Figure 2b).

#### 3.2.1 Intramuscular Administration

Intramuscular administration has emerged as the primary delivery route for mRNA vaccines due to its localized pharmacological effects. Post‐immunization analyses reveal that LNPs elicit innate immune activation, characterized by monocyte and dendritic cell recruitment to the injection site. This cellular infiltration is associated with localized inflammation, manifested as pain, swelling, and fever [98, 108, 125]. Mechanistic investigations demonstrate that this immunogenicity arises from TLR pathway activation following LNP endocytosis by APCs. Comparative studies indicate that poly(amine‐co‐ester)‐based polymer vectors achieve 7.7‐fold higher protein expression than LNPs following intramuscular delivery, while paradoxically inducing reduced inflammatory reactions [136]. This suggests that cationic polymers may employ distinct endocytic mechanisms to circumvent TLR recognition. It is worth noting that the selection of lipid components and administration routes requires careful optimization to balance immunogenic potency with acceptable tolerability profiles. Intramuscular injection represents the optimal administration route, demonstrating superior enhancement of both antibody production and T cell responses. In contrast, intravenous administration induces elevated systemic inflammation as evidenced by increased expression of IL‐6 and monocyte chemotactic protein (MCP)‐1, though concurrent IL‐10 upregulation may counteract immune activation [51]. Structural refinement of lipid components can improve local tissue retention while minimizing systemic biodistribution, thereby mitigating inflammation‐related risks. For example, compared with the DLin‐MC3‐DMA/mRNA formulation, the DOG‐IM4/mRNA system exhibited delayed and attenuated inflammatory responses, including reduced febrile reactions, neutrophil recruitment, and inflammatory mediators (e.g., CCL‐2, IL‐6) [125]. Such engineering advances underscore the pivotal equilibrium between immunogenicity and safety in advancing next‐generation mRNA delivery platforms.

#### 3.2.2 Intravenous Administration

Intravenous administration serves as the predominant systemic delivery method for LNPs, enabling rapid biodistribution to immune organs such as the liver and spleen, where they trigger explosive elevation of proinflammatory cytokines. The inflammatory cascade induced by intravenous LNPs involves multifaceted mechanisms encompassing complement activation, platelet activation, and thrombotic risks. Cationic lipid‐based LNPs exhibit high affinity to plasma fibrinogen, promoting platelet activation and coagulation cascades that contribute to pulmonary embolism and systemic inflammation [42]. Furthermore, the inherent liver–spleen targeting effect of LNPs may activate Kupffer cells, which subsequently release proinflammatory mediators via NLRP3 inflammasome activation [77, 137]. To address these side effects, recent strategies have focused on redirecting LNP tropism. N‐acetyl‐D‐galactosamine (GalNAc) ligand‐modified LNPs demonstrate preferential targeting to hepatocytes over immune cells, thus reducing systemic inflammatory responses [77]. While intravenous delivery of retinoic acid‐inducible gene I agonist‐encapsulated LNPs enhances CD8^+^ T cell infiltration in tumor microenvironments, dose‐dependent cytokine storm risks remain a critical limitation [138]. Mechanistic studies highlight the dual roles of PEGylation in modulating immune interactions: high PEG surface density reduces monocyte phagocytosis but paradoxically exacerbates inflammation through alternative complement pathway activation [98]. Emerging engineering approaches utilize the theory of “ESCRT‐repairable endosomal damage” to refine intravenous LNPs. In this aspect, incorporating rapidly degradable ionizable lipids, such as 4A3‐SC8, minimizes irreversible endosomal membrane disruption, achieving over 50% reduction in inflammatory responses [47]. This innovation indicates the importance of balancing endosomal escape efficiency with membrane repair kinetics to optimize therapeutic safety. Collectively, these advances highlight the potential of structure–function‐guided LNP design to decouple immunostimulatory efficacy from systemic toxicity for intravenous mRNA therapeutics.

#### 3.2.3 Respiratory Administration

Respiratory delivery offers distinct pharmacokinetic advantages by circumventing hepatic first‐pass metabolism. LNPs administered via the respiratory mucosa demonstrate unique immunomodulatory characteristics. Alveolar macrophage‐mediated endocytosis primarily controls localized inflammatory responses, where particle size critically determines cellular uptake patterns. LNPs smaller than 100 nm exhibit superior mucus penetration and preferential uptake by epithelial cells [139]. However, larger particles are mainly phagocytized by macrophages, which activate the NF‐κB signaling pathway through the TLR4/MyD88 pathway [140]. Animal studies reveal that intranasal delivery achieves high transfection efficiency in pulmonary epithelial cells, generating systemic inflammatory cytokine levels merely 20% of those observed with intravenous administration [136]. This attenuated immunogenicity may stem from mucosal tolerance mechanisms, characterized by reduced phagocytic activity of alveolar macrophages and elevated activation thresholds for TLR signaling in the respiratory mucosa. However, repeated administration may disrupt mucosal barrier function, potentially exacerbating local neutrophil infiltration. Notably, intranasally delivered LNPs induce three‐fold higher IL‐10 production relative to intramuscular delivery, a response mediated by the abundant TLR7/8 expression in pulmonary tissues [136]. Formulation stability presents a key translational hurdle, as aerosolization‐induced shear force compromises LNP structural integrity. Recent advances demonstrate that incorporation of cholesterol derivatives improves mechanical stability, mitigating both endosomal membrane disruption and downstream pro‐inflammatory cascades [141, 142, 143]. These developments highlight the critical balance required between enhancing LNP bioactivity and controlling pulmonary immunotoxicity, providing key design principles for future inhaled mRNA therapeutics.

#### 3.2.4 Intraperitoneal Administration

Intraperitoneal injection enables rapid systemic absorption, often eliciting transient yet intense peritoneal inflammatory responses. Unlike other administration routes, intraperitoneally delivered LNPs exhibit distinct biodistribution patterns, showing broader tissue dissemination and preferential accumulation in Kupffer cells and peritoneal resident macrophages [136]. This triggers a unique cytokine profile characterized by concurrent IL‐10 and IL‐12 secretion, with rapid upregulation of IL‐12 and IFN‐γ following administration [138]. Such acute inflammatory responses may enhance antitumor immunity in immunotherapeutic applications, demonstrating a three‐fold increase in CD8^+^ T cell activation without inducing systemic inflammation [138]. Mechanistically, the localized immune activation stems from LNP interactions with PRRs on peritoneal macrophages, which coordinate Th1‐polarized immune responses while maintaining regulatory cytokine production. However, chronic disease models uncover potential adverse effects, particularly the development of peritoneal adhesions, emphasizing the necessity for rigorous clinical indication evaluation. These contrasting outcomes highlight the critical role of administration context: acute intraperitoneal delivery exploits transient inflammatory responses for immune stimulation, while chronic exposure may lead to fibrotic complications.

The preceding sections have systematically outlined the inflammatory features of LNPs across different administration routes. However, much of this knowledge remains at the level of phenomenological description, and our mechanistic understanding of how regional immune microenvironments fundamentally shape these differences is still quite limited. Taking intramuscular injection as an example: while existing studies have established that this route recruits neutrophils and activates dendritic cells, what remains unclear is how these two innate immune cell populations coordinate with the draining lymphatic system, and how local signals are integrated and relayed to the lymph nodes to modulate adaptive immune responses, questions that have rarely been explored mechanistically. In the case of intravenous administration, the interplay among complement activation, platelet aggregation, and the coagulation cascade in LNP‐induced systemic inflammation remains poorly understood from an integrated perspective, despite accumulating evidence suggesting that these pathways may collectively amplify inflammatory responses. Moreover, most current studies rely on endpoint measurements, such as cytokine levels at a single time point, to assess inflammatory status. Such approaches fall to capture the spatiotemporal dynamics of immune activation across different routes and limit our ability to determine whether the duration of inflammation, rather than its peak magnitude, correlates with therapeutic outcomes or adverse effects. From a translational perspective, significant differences exist between rodents and humans in tissue anatomy, immune cell composition, and lymphatic drainage patterns, meaning that route‐specific findings derived from mouse models may not be directly extrapolated to humans. For instance, the intramuscular space in mice differs from that in humans in terms of muscle fiber type, vascularization, and resident immune cell populations. These differences may profoundly impact LNP distribution and local inflammatory responses. Addressing these gaps requires moving beyond descriptive studies toward mechanistic integration, leveraging approaches such as high‐resolution imaging of immune cell dynamics, multi‐omics profiling, and human‐relevant models to establish a predictive framework for route‐specific LNP design.

### 3.3 Regulation of Inflammatory Responses by Tissue‐Specific Biodistribution

The biodistribution characteristics of LNPs determine the organ‐specificity and intensity of their inflammatory responses, which are controlled by dynamic interactions between innate immune cells and local microenvironmental factors. Due to their intrinsic liver tropism, LNPs predominantly accumulate in hepatic tissues through passive targeting mechanisms, making liver‐mediated responses a key determinant of systemic inflammation risk. This hepatic targeting is particularly evident in systemically delivered mRNA‐LNPs, whose liver specificity is mediated by apolipoprotein E (ApoE) binding and subsequent recognition by ApoE receptors on the surface of hepatocytes (Figure 2c). Notably, modulating LNP lipid composition enables differential targeting of hepatic cell populations, including hepatocytes, Kupffer cells, and endothelial cells, by altering ApoE affinity or activating non‐ApoE pathways (e.g., lipoprotein receptor‐associated protein 1 (LRP1)‐ and albumin receptor‐mediated macropinocytosis) [144, 145, 146, 147]. In the hepatic microenvironment, Kupffer cells lining the sinusoids serve as the primary site of innate immune activation, orchestrating potent cytokine production. Inflammatory responses of liver‐targeted LNPs exhibit dual dependency on both the administered dose and lipid composition. Notably, MC3 lipid‐formulated LNPs can enhance the delivery efficiency of STING agonists to hepatic leukocytes, resulting in type I IFN pathway activation and initiation of liver‐specific inflammatory cascades [79]. These findings collectively position liver‐targeting modifications as tunable molecular switches for precise inflammation modulation.

Non‐hepatic LNP systems display distinct organ‐specific inflammatory profiles (Figure 2c). Unmodified LNPs typically induce hepatic cytokine release (IL‐1β and MCP‐1), while modifications, such as anti‐PECAM‐1 antibody and GalNAc ligand conjugation, significantly reduce hepatic retention and subsequent systemic inflammation [77, 148]. Cationic LNPs show preferential lung accumulation but require PEGylation or lipid composition optimization to reduce nonspecific protein adsorption and inflammatory responses. By replacing conventional amino lipids with cationic sulfonium species, the engineered LNP platform composed of novel sulfonium lipids and accessory components enables efficient lung‐specific mRNA delivery with attenuated immunogenicity, mainly by precise optimization of endothelial targeting while avoiding immune cell recognition [149]. Notably, lung‐targeted ZP‐LNPs employ zeta‐potential engineering to circumvent Kupffer cell clearance, enabling effective siTNF‐α delivery that specifically reduces pulmonary TNF‐α levels in inflammatory models without altering hepatic inflammatory (TNF‐α, IL‐6) or injury biomarkers (ALT, AST) [105]. Similarly, orally administered LNPs targeting colonic macrophages via asialoglycoprotein receptor rebalanced the local IL‐6/IL‐10 cytokine ratio. Through restoration of the mucosal barrier integrity, these LNPs achieved localized suppression of colonic inflammation while preventing systemic inflammatory responses [69]. The intrathecal delivery of PU.1 siRNA via MG‐LNPs capitalizes on the immune‐privileged microenvironment of the central nervous system, where the blood–brain barrier (BBB) restricts both peripheral immune cell infiltration and systemic drug distribution. This delivery approach allows direct modulation of microglial activity while circumventing off‐target effects. On the other hand, VCAM‐1‐targeted LNPs can efficiently deliver therapeutic mRNAs (e.g., thrombomodulin mRNA) specifically to inflamed cerebrovascular endothelial cells, thereby reinforcing the BBB integrity and mitigating cerebral edema [150, 151]. Nevertheless, non‐hepatic LNPs face an immunological paradox: intraocular administration of LNPs achieves efficient Müller cell transfection without inducing IL‐6 elevation or microglial activation within 6 h [152], while intravenous injection results in widespread biodistribution (particularly to the liver and spleen) and triggers systemic inflammation characterized by elevated serum levels of IFN‐α, TNF, and CRP. This systemic response is further evidenced by dose‐dependent weight loss and increased cardiac injury markers [121]. This dichotomy underscores administration route‐dependent immune surveillance: localized delivery bypasses systemic immune recognition, while intravenous administration necessitates surface engineering strategies (e.g., PEGylation) to minimize opsonization and subsequent immune detection [71]. Within the intravenous route, however, the organ distribution pattern further dictates the balance between therapeutic efficacy and reactogenicity. Although intravenous injection distributes LNPs to both the liver and spleen, only splenic protein expression correlates positively with the magnitude of immune responses and the severity of adverse reactions (e.g., fever, elevated IL‐6 and TNF‐α levels); hepatic expression shows no such correlation [59]. These findings position splenic protein expression as a potential screening marker for low‐reactogenicity LNP formulations [59], suggesting that non‐hepatic targeting strategies should consider splenic exposure when aiming to decouple therapeutic efficacy from reactogenicity.

## 4 Strategies to Regulate Inflammation

### 4.1 Modulation of Physicochemical Properties of LNPs

**FIGURE 3:**
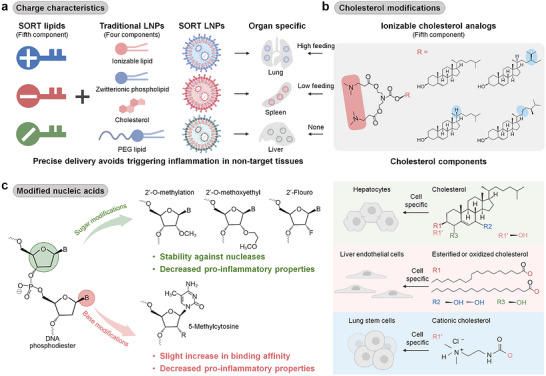
Modulation of physicochemical properties of LNPs to regulate inflammatory responses. (a) Modulation of charge characteristics. Incorporating a fifth charge‐modulating lipid (e.g., SORT molecules) enables programmatic tuning of surface charge and endosomal escape kinetics in classical four‐component LNPs. This dual‐parameter control not only redirects in vivo biodistribution for extrahepatic organ targeting but also fundamentally attenuates immunostimulatory potency by modulating LNP‐endosomal membrane interactions, thereby mitigating off‐target inflammatory responses. (b) Cholesterol modifications. Chemical engineering of cholesterol, either through covalent modification or replacement with structural analogs as a fifth component, enables precise control of LNP membrane properties and organelle‐specific interactions. This strategy reprograms intracellular trafficking (e.g., circumventing NLRP3 inflammasome activation) while enhancing target specificity, thereby mitigating inflammation from nonspecific membrane damage without compromising delivery efficiency. (c) Modified nucleic acids. Chemical modification of nucleic acids represents the primary strategy to abrogate innate immunogenicity. Through either nucleoside replacement (e.g., pseudouridine) or ribose engineering, these alterations minimize intracellular PRR recognition, thereby effectively decoupling immune activation from coding/silencing functionality while preserving therapeutic efficacy.

Strategic optimization of LNP physicochemical properties represents a powerful approach to mitigate inflammatory responses. Precise engineering of surface characteristics through potential modifications, coupled with judicious selection of cholesterol derivatives, enables organ‐ and cell‐specific targeting that minimizes off‐target inflammation. Concurrently, structural refinement of nucleic acid payloads through chemical modification or sequence optimization significantly reduces their intrinsic immunogenicity, thereby attenuating systemic inflammatory cascades. These rationally designed modifications collectively establish a robust framework for developing inflammation‐regulatable LNP formulations (Figure 3).

#### 4.1.1 Charge Characteristics

The strategic optimization of lipid formulations holds paramount importance in achieving precise anti‐inflammatory effects through organ‐specific targeting and microenvironmental regulation (Figure 3a). By adjusting charge characteristics, cholesterol composition, and other physicochemical properties of LNPs, their organ targeting ability can be significantly enhanced while simultaneously modulating inflammatory responses. Studies have shown that LNP surface charge directly governs biomembrane interactions and in vivo distribution patterns. Positively charged LNPs are more likely to be captured by immune cells, while neutral or negatively modified particles exhibit reduced nonspecific clearance and improved targeting efficiency to organs like the spleen [153, 154, 155]. This understanding has enabled the development of selective organ targeting (SORT) technology, which utilizes charge‐based “localization molecules” to precisely direct LNPs to hepatic, pulmonary, or splenic tissues. Charge differences lead to differential electrostatic adsorption of mRNA to the vascular endothelium of specific tissues [156]. The SORT platform successfully reconciles organ‐specificity with controlled immunogenicity without compromising mRNA delivery efficiency [146, 156]. A representative application involves lung‐targeting LNPs that combine cationic phospholipids with optimized zeta‐potential, thus reducing serum protein binding while enhancing fusion competence with alveolar epithelial cell membranes [42, 157].

#### 4.1.2 Cholesterol Modifications

Cholesterol functions as the fundamental structural component of LNPs, with its chemical modifications critically determining targeting specificity (Figure 3b). Native cholesterol preferentially directs hepatic accumulation by mimicking low‐density lipoprotein metabolic pathways, while structural variants such as esterified or oxidized cholesterol derivatives alter biodistribution patterns through different lipoprotein‐mediated transport mechanisms. Incorporation of these modified cholesterol forms enables LNPs to emulate endogenous lipoprotein behaviors and modulate their binding affinity for various lipoprotein receptors [147]. In addition, the hydroxylation state of cholesterol further affects LNP metabolic processes in macrophages, where a reasonable selection of cholesterol derivatives can reduce lysosomal retention and subsequent pro‐inflammatory cytokine release [57]. Notably, cationic cholesterol derivatives exhibit unique properties compared to conventional helper lipids. When paired with neutral accessory lipids, cationic cholesterol maintains dual organotropsm capable of mediating efficient mRNA delivery to both hepatocytes and pulmonary tissues. Strategic combination with cationic auxiliary lipids enables precise redirection of LNP biodistribution, achieving cell‐type‐specific targeting of cardiac endothelial cells and lung stem cells [158]. The charged state of cholesterol can alter the biodistribution profiles of LNPs by regulating their interactions with cell membranes, endocytosis pathways, and serum protein adsorption. This indicates that cholesterol functions not only as a key structural stabilizer but also as a tunable element for tissue‐specific mRNA delivery. The discovery of this charge‐dependent targeting mechanism provides new insight into engineering multi‐organ targeted gene delivery systems. For example, pH‐responsive di‐tertiary amine cholesterol derivatives have been developed as fifth components to precisely control LNP surface charge. At lower incorporation ratios (10%–30%), these derivatives enhance β2‐glycoprotein I absorption and enable 95% splenic targeting. By contrast, higher ratios (40%–50%) increase zeta‐potential to induce particle aggregation and lung‐affinity protein corona formation, shifting biodistribution to 78% pulmonary targeting. Remarkably, this charge‐switching principle maintains consistent behaviors across diverse lipid systems, including ALC‐0315, SM‐102, and U‐101, demonstrating the broad applicability of this strategy [159].

#### 4.1.3 Modified Nucleic Acids

The modification of nucleic acids (such as siRNA) can achieve dual therapeutic functionality by combining targeted gene silencing with precise inflammatory pathway regulation, thereby reducing the immunogenicity of LNPs through controlled modulation of inflammation‐associated genes (Figure 3c). In siRNA‐based strategies targeting inflammatory mediators, TAK1 kinase‐specific siRNA has been shown to substantially attenuate pro‐inflammatory responses in macrophages by blocking both NF‐κB and MAPK signaling pathways, though such approaches require cell‐specific targeting ligands (e.g., F4/80 antibodies) to ensure cellular selectivity [160]. The strategic incorporation of chemically modified nucleic acids offers enhanced immunomodulatory potential, where 2′‐O‐methylation of oligonucleotide sequences has proven particularly effective in mitigating TLR7/8‐mediated innate immune activation [90, 161, 162]. Parallel advances in miRNA therapeutics are exemplified by miR‐29a delivery systems, which demonstrate dual‐pathway inhibition of both NF‐κB signaling and NLRP3 inflammasome activation, yielding coordinated anti‐inflammatory and tissue‐regenerative effects in tendon repair models [163]. Recent studies demonstrate that engineered dsRNA adjuvants, exemplified by ARNAX, selectively target TLR3 in dendritic cells, thus significantly reducing systemic inflammatory responses while preserving vaccine efficacy [164]. These findings underscore the critical balance required between targeting specificity and immune‐silencing capacity in nucleic acid design [165, 166, 167]. Future development should prioritize multi‐target intervention approaches, such as simultaneous inhibition of PGE2 and NLRP3 inflammasome pathways, to amplify synergistic anti‐inflammatory outcomes while maintaining therapeutic precision.

### 4.2 Optimization of the Chemical Structure of Lipids

**FIGURE 4:**
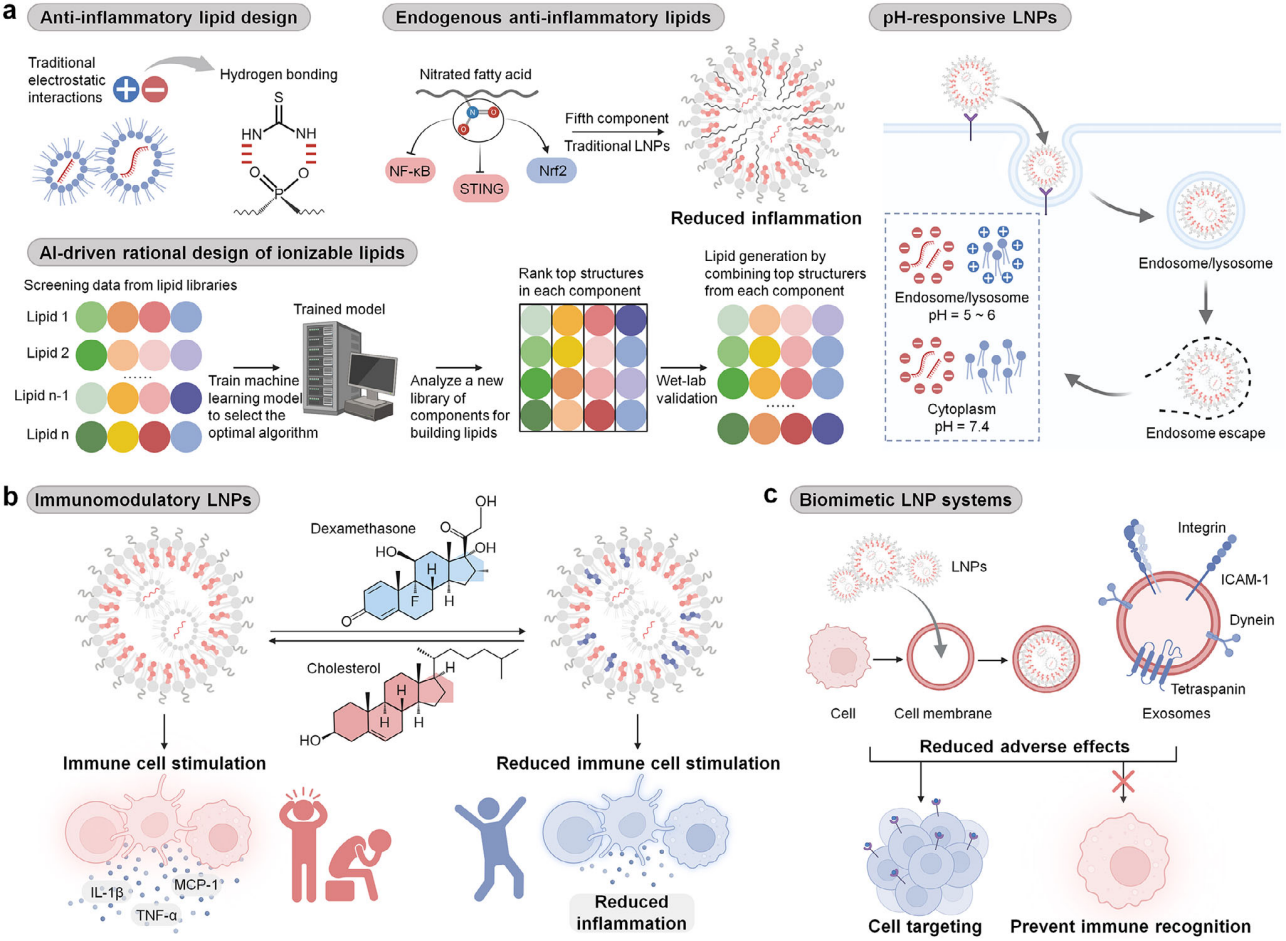
Strategies to regulate inflammation induced by LNPs. (a) Optimization of lipid chemical structures. LNPs with intrinsic anti‐inflammatory properties are designed from molecular sources to be rapidly degraded upon completion of endosomal escape to limit sustained immune activation. Endogenous anti‐inflammatory lipids are introduced directly into the formulation. Develop “smart” LNPs that respond to pathological microenvironments, such as low pH, to achieve on‐demand release of inflammatory signals. Using AI‐driven lipid screening platforms, identify molecules with the optimized delivery efficiency‐to‐immunogenicity ratio. (b) Design of immunomodulatory LNPs. LNPs are engineered as immunomodulatory co‐delivery systems by structurally integrating anti‐inflammatory small molecules. For example, partial substitution of cholesterol with dexamethasone creates bifunctional LNPs that concurrently deliver nucleic acid payloads and suppress local innate immunity, thus achieving source‐level mitigation of inflammatory sequelae while creating therapeutic synergy. (c) Biomimetic LNP systems. Biological interface engineering confers “stealth” and targeting capabilities to LNPs. Excellent biocompatibility can be afforded to LNPs by coating with native cell membranes or exosomes. This biomimetic camouflage effectively evades immune recognition/clearance, and exploits source cell‐derived pathotropism for tissue‐specific delivery, thereby minimizing off‐target interactions and subsequent inflammation.

According to existing studies, strategic structural optimization of ionizable lipids combined with anti‐inflammatory lipid components can substantially enhance both the safety profile and inflammatory response characteristics of LNP‐based nucleic acid delivery systems (Figure 4a). As the core functional components of LNPs, ionizable lipids directly affect nucleic acid encapsulation efficiency, endosomal escape capability, and immunogenic potential. Comparative studies reveal significant differences in inflammatory responses between clinically approved formulations. SM‐102 (utilized in Moderna's COVID‐19 vaccine mRNA‐1273) exhibits pronounced immunogenicity, while DLin‐MC3‐DMA (from Alnylam's FDA‐approved siRNA therapeutic Patisiran) demonstrates relatively inert behaviors. This discrepancy appears to originate from structure‐specific interactions between their amine headgroups and immune PRRs, particularly TLR4 and CD1d [168]. Recent advances in anti‐inflammatory lipid design have yielded promising strategies for mitigating innate immune activation. Notably, β‐aminoester lipids, synthesized by Michael addition of acrylate to diethanolamine followed by fatty acid esterification, can effectively inhibit type I interferon pathway activation and reduce pro‐inflammatory cytokine production during self‐amplifying RNA delivery [169]. Although mRNA delivery via classical cationic lipids has advanced significantly, their requisite high cationic charge density for mRNA condensation inevitably induces inflammation and cytotoxicity. To address this issue, non‐cationic thiourea LNPs (NC‐TNPs) were developed, featuring thiourea groups as hydrogen‐bond donors and T‐shaped hydroxyl groups as supplementary bond donors. This system represents a paradigm shift from traditional electrostatic‐driven mRNA‐cationic lipid assembly by leveraging strong hydrogen bonding between NC‐TNP thiourea moieties and mRNA phosphate groups. NC‐TNPs demonstrate enhanced mRNA‐encoded protein expression and exhibit negligible inflammatory/cytotoxic effects in vivo, outperforming conventional cationic or ionizable mRNA‐LNPs with charge‐associated safety concerns [170]. These findings establish a framework for developing next‐generation ionizable lipids with optimized therapeutic indices through balanced consideration of delivery efficiency and immunomodulatory properties.

#### 4.2.1 pH‐Responsive LNPs

Recent studies have revealed substantial advancements in precision nucleic acid delivery and inflammation control through sophisticated LNP engineering incorporating microenvironment‐responsive release strategies. These optimized LNP formulations leverage pH‐dependent properties to achieve spatiotemporal control of nucleic acid release via charge reversal or structural transformation upon exposure to acidic microenvironments, such as lysosomes and inflamed tissues. The rational design of ionizable lipids enables LNPs to remain neutral at physiological pH to minimize nonspecific uptake, while undergoing protonation in endosomal acidic conditions (pH 4.5–6.5), thereby facilitating efficient endosomal escape and therapeutic payload release (Figure 4a) [171, 172]. For example, incorporation of cationic amphiphilic drugs as a fifth component in LNPs can optimize siRNA and mRNA cytosolic delivery efficiency through microfluidic engineering. Their ionizable properties may promote membrane fusion or facilitate structural remodeling in acidic endosomal environments, thereby enhancing nucleic acid release [173]. Furthermore, auxiliary lipids like DOTAP can enhance tissue‐specific targeting by modulating LNP surface charge under defined pH conditions. This strategy enables efficient mRNA delivery in organs such as the lungs and liver [174]. These systems exhibit particular promise in inflammatory or tumor microenvironments characterized by lower pH. For instance, optimizing pH‐responsive features of LNPs in lung cancer models reduces systemic toxicity while enhancing localized therapeutic gene expression [175]. Hydrophobic scaffolds with long chains, linear pH‐sensitive cationic lipids can significantly improve the siRNA delivery efficiency of small LNPs by reducing lipid diffusion and improving endosomal escape [176]. However, a key challenge remains in balancing delivery efficiency with the risk of nucleic acid degradation following endosomal escape. Current research highlights the need to refine pH‐triggered release kinetics and lipid composition to ensure precise payload release [78]. Such advancements emphasize the importance of integrating materials science with biological insights to develop next‐generation LNPs for precision medicine.

#### 4.2.2 Endogenous Anti‐Inflammatory Lipids

Furthermore, incorporating endogenous anti‐inflammatory lipids (e.g., polyunsaturated fatty acid derivatives) can synergistically regulate the immunometabolic pathways of LNPs. These bioactive lipids suppress systemic inflammatory responses by inhibiting the activation of NF‐κB and the inflammasome, thereby reducing the secretion of pro‐inflammatory factors such as IL‐6 and TNF‐α. For example, in mRNA vaccine design, the combination of ionizable lipids with oleic acid reduced the incidence of local redness and vascular leakage in a mouse model while maintaining equivalent immunogenicity [177]. Brenner's group developed an innovative solution to mitigate severe inflammatory responses induced by pDNA delivered through LNPs. Their engineered system (NOA‐pDNA‐LNPs) incorporates endogenous anti‐inflammatory lipids like nitrooleic acid (NOA) [178]. This platform achieved both enhanced safety and sustained transgene expression by inhibiting the cGAS‐STING pathway (Figure 4a). The NOA‐pDNA‐LNPs platform demonstrates prolonged therapeutic gene expression, minimized toxicity, and modular optimization capability. These features establish it as a versatile platform for gene therapy, vaccine development, and CRISPR‐based genome editing. Importantly, it addresses the critical unmet need for effective non‐viral delivery systems for the treatment of chronic diseases.

#### 4.2.3 Artificial Intelligence (AI)‐Driven Rational Design of Ionizable Lipids

AI platforms have revolutionized LNPs development by addressing the limitations of conventional screening methods, including low efficiency and prohibitive costs [168, 179, 180]. These computational approaches facilitate high‐throughput virtual screening of lipid libraries, rational design of novel ionizable lipids, and optimization of LNP formulations for enhanced targeting specificity and biocompatibility (Figure 4a). A notable example is the Triplet LNP platform, developed through AI‐guided lipid library screening, which enables co‐delivery of multiple immunomodulatory payloads, including cytokines and T‐cell activators. This system significantly enhances CD8^+^ T‐cell infiltration and antitumor activity in immunosuppressive microenvironments [181]. Concurrently, advanced lipid engineering has yielded β‐aminoester lipids and C12‐200 derivatives. These materials permit control of LNP immunogenicity, allowing tailored inflammatory responses to meet specific therapeutic requirements [51, 169].

### 4.3 Design of Immunomodulatory LNPs

Immunomodulatory LNPs represent a versatile therapeutic platform capable of simultaneously addressing inflammation through direct anti‐inflammatory action via co‐encapsulated pharmacological agents and microenvironmental reprogramming via targeted nucleic acid delivery to restore immune homeostasis under pathological conditions. This combinatorial approach not only suppresses acute inflammatory responses but also fundamentally alters the disease microenvironment, as demonstrated in various inflammatory disease models (Figure 4b). The synergistic integration of small molecule therapeutics with genetic modulators within a single LNP system enables spatiotemporal coordination of immediate anti‐inflammatory effects with long‐term immune resetting, offering unprecedented precision in inflammation regulation.

#### 4.3.1 Co‐Delivery of Anti‐Inflammatory Agents and Nucleic Acids

Formulation optimization of LNPs to incorporate anti‐inflammatory drugs offers a promising approach for synergistic co‐delivery of nucleic acid therapeutics and anti‐inflammatory agents, thereby mitigating inflammatory responses. A prime example involves the development of DSPC‐LNP and DOPE‐LNP formulations, in which 25% of cholesterol was replaced with dexamethasone, a representative anti‐inflammatory drug. These engineered LNPs not only enabled efficient delivery of reporter mRNA but also significantly reduced levels of pro‐inflammatory cytokines (such as IL‐1β, TNF‐α, and MCP‐1) [182, 183]. In vivo studies demonstrated that this co‐delivery strategy enhanced mRNA expression by 1.5‐fold while avoiding the inflammatory cascade typically associated with conventional LNPs [183]. Similarly, co‐encapsulation of lipid‐conjugated dexamethasone with mRNA in LNPs suppressed TLR signaling‐mediated inflammation, leading to prolonged protein expression in the liver [184]. Furthermore, pretreatment with Janus kinase (JAK) inhibitors or glucocorticoids has been shown to effectively alleviate cytokine storm and associated organ injury caused by LNP‐encapsulated siRNA, without compromising its gene silencing activity [185]. These pharmacological approaches exert rapid anti‐inflammatory effects by targeted modulation of key signaling pathways, particularly NF‐κB and JAK‐STAT. Collectively, the rational design of LNPs for combinatorial delivery of nucleic acids and anti‐inflammatory agents provides a promising avenue to enhance therapeutic outcomes while minimizing adverse inflammatory complications [186].

#### 4.3.2 Applications of Immunomodulatory LNPs for Microenvironment Reprogramming

The immunogenic profile of LNPs can be precisely modulated through formulation engineering. For instance, DSPS‐containing LNPs demonstrate selective tropism for myeloid cells (such as neutrophils and macrophages) while attenuating lymphocyte activation, thereby reducing systemic inflammatory risks [187]. Furthermore, endogenous extracellular vesicle‐encapsulated LNP components exhibit significantly suppressed cytokine induction compared to conventional LNPs, suggesting their particular advantage for chronic therapeutic applications [72]. Together, these advances in LNP engineering, encompassing enhanced targeting specificity, novel biomaterial development, and precise immunomodulation, create a robust framework for developing next‐generation immunomodulatory nanotherapeutics. By synergistically bridging nucleic acid delivery with immune microenvironment reprogramming, these innovative strategies hold transformative therapeutic potential across a spectrum of clinical applications, including inflammatory diseases, neurological disorders, and malignancies.

### 4.4 Biomimetic LNP Systems

Biomimetic engineering approaches employing natural membrane coatings, such as cell‐derived membranes and exosomal vesicles, confer LNPs with enhanced immune evasion capabilities and tissue‐specific targeting precision, while simultaneously mitigating off‐target effects. These biologically inspired camouflage strategies leverage endogenous trafficking mechanisms to achieve superior biodistribution profiles, as they effectively mimic native cellular components to bypass immune surveillance systems. The resulting reduced systemic toxicity and improved therapeutic index establish biomembrane‐functionalized LNPs as a promising platform to develop nucleic acid nanotherapies for diverse diseases (Figure 4c).

#### 4.4.1 Cell Membrane‐Derived Biomimetic Delivery Systems

Biomimetic LNP technology has significantly improved targeted nucleic acid delivery while mitigating systemic inflammatory side effects. For example, hybridizing natural cell membranes with liposomal bilayers integrates diverse biological functions, such as enhanced targeting and immune evasion. Specifically, integrating tumor cell membranes with stimuli‐responsive liposomes enables targeted delivery of phosphoglycerate mutase 1 siRNA to regulate inflammatory microenvironments, providing a precise combinatorial strategy with chemotherapy for non‐small cell lung cancer [188]. Alternatively, biomimetic LNPs incorporating the myoblast fusion regulator 1,2‐dioleoyl‐sn‐glycero‐3‐phospho‐(1′‐myo‐inositol‐4′,5′‐bisphosphate) have been developed to mimic fusogenic membrane structures for muscle‐specific targeting. This platform utilizes sucrose co‐delivery to transiently inhibit lysosomal function in target tissues, thereby enhancing intracellular nucleic acid stability. Based on this strategy, DNA vaccination in mouse muscle demonstrates significantly prolonged viral antigen expression without inducing systemic inflammation, toxicity, or elevation of serum inflammatory factors (IL‐6, IFN‐γ) [25].

#### 4.4.2 EV‐Based Biomimetic Delivery Systems

EVs, naturally secreted by cells, offer unique advantages as biomimetic drug carriers due to their inherent biocompatibility and functional versatility. The membrane architecture of EVs, enriched with bioactive molecules such as proteins, nucleic acids, and lipids, confers low immunogenicity and prolonged circulation by evading immune surveillance, thereby enhancing systemic retention [73]. Compared to synthetic LNPs, EVs exhibit superior cellular recognition and internalization efficiency owing to their endogenous origin. Bioengineered LNPs incorporating biomimetic membrane components, for instance, retain conventional LNP benefits while acquiring enhanced biocompatibility through natural membrane‐derived motifs [189]. In angiogenic therapies, VEGF‐A mRNA delivered by LNPs is partially secreted into EVs following cellular uptake. These EVs amplify therapeutic outcomes through secondary distribution of pro‐angiogenic transcripts such as matrix metalloproteinase‐2/14 and angiopoietin‐like 4, establishing a cascaded delivery mechanism [73]. EVs further enable precise microenvironmental regulation via intrinsic signaling pathways. Engineering strategies such as membrane modification can enhance tissue specificity and synergize therapeutic effects through fusion of targeting ligands or immunoregulatory molecules [73, 189]. Notably, combinatorial approaches integrating EVs with LNPs demonstrate translational promise: LNP‐mediated mRNA delivery can use EVs for secondary biodistribution, prolonging transgene expression while minimizing systemic toxicity [73].

### 4.5 Finding the Optimal Balance in Practice

While the strategies described in previous sections each offer distinct advantages for mitigating LNP‐induced inflammation, no single approach is universally optimal. Every modification intended to reduce inflammation inevitably introduces trade‐offs, most commonly diminished delivery efficiency, increased manufacturing complexity, or compromised targeting precision. Table 3 presents a side‐by‐side comparison of these strategies across key parameters, highlighting their respective strengths and limitations.

**TABLE 3:** Strategies for regulating LNP‐induced inflammation.

Strategies	Anti‐inflammatory effects	Possible negative effects on transfection efficiency	Challenges in scalable manufacturing	Targeting specificity	Key limitations	References
Charge modulation	Moderate to high; Reducing non‐specific immune uptake	Low to moderate; Risk of over‐neutralization reducing endosomal escape	Low; Adjustable via lipid composition	High; Organ‐selectivity	Immune cell capture and reduced in vivo stability associated with positively charged LNPs; Reduced cationic charge may decrease endosomal escape efficiency	[153, 157, 174]
Cholesterol modification	Moderate; Reducing lysosomal retention	Low to moderate; Derivative‐dependent effects	Moderate; Synthesis of derivatives required	High; Tunable organ tropism	Unexpected metabolic effects from certain oxidized/esterified cholesterol forms; Long‐term stability requires validation	[31, 59, 141, 147, 158]
Nucleic acid modification	High; Direct silencing or inhibition of immune pathways	Low; Preservation of function via chemical modifications	Low; Standard solid‐phase synthesis	Low to moderate; Dependent on additional targeting ligands	Requires cell‐specific targeting ligands for selectivity; Off‐target gene silencing risk; Increased synthesis cost	[160, 258, 259]
Ionizable lipid structure optimization	High; Negligible inflammation (e.g., thiourea LNPs)	Low; Improved escape and enhanced expression	High; Novel lipid synthesis and purification	Moderate; Inherent tropism, but requires tuning	Extensive safety profiling required; Sensitivity to formulation conditions (e.g., hydrogen‐bonding systems)	[169, 170, 258, 260, 261, 262, 263]
pH‐responsive LNPs	Moderate; Reducing non‐specific uptake at physiological pH	High; Risk of payload leakage or degradation with poor optimization	Moderate; Microfluidic mixing required	Moderate to high; Acidic tissue targeting	Balancing endosomal escape with premature release	[46, 260]
Endogenous anti‐inflammatory lipids	High; Suppressing NF‐κB and cGAS‐STING pathways	Low to moderate; Slight structural alteration from co‐delivery	Moderate; Incorporation into standard formulations	Low; Not inherently targeted	Potential interference with long‐term stability	[178]
AI‐driven design of ionizable lipids	High; Optimization for low immunogenicity	Low; Prediction of high‐efficiency candidates	High; Synthesis of novel lipids at scale	High; Tailorable for specific organs	High‐quality training data required; Predicted properties require experimental validation; Computational cost	[180, 261, 264, 265]
Co‐delivery of anti‐inflammatory agents	High; Direct pharmacological suppression	Low; Enhanced mRNA expression via co‐delivery	Low to moderate; Simple co‐encapsulation	Low; Dependent on additional targeting ligands	Potential drug‐lipid interactions affecting long‐term stability; Risk of systemic immunosuppression upon off‐target release	[182, 183, 199]
Immunomodulatory LNPs	Moderate to high; Myeloid‐selective or EV‐camouflaged	Low; Improved delivery in some designs	High; Complex EV isolation and engineering	High; Cell‐type selective	Low‐yield endogenous extracellular vesicle production; Scale‐up and quality control challenges	[72, 187, 258]
Biomimetic LNPs	High; Immune evasion via “self” markers	Low; Preservation of core delivery via camouflage	Very high; Membrane coating, extrusion, characterization	High; Source‐cell dependent	Batch‐to‐batch variability; Coating integrity during storage; Potential antigenicity of membrane proteins	[188, 189, 266]

The key limitations summarized in Table 3 underscore that there is no one‐size‐fits‐all solution. The optimal strategy depends critically on the therapeutic context. For vaccine applications, where transient inflammation is desirable as an adjuvant effect, a low‐to‐moderate inflammatory profile may be acceptable. However, achieving potent and durable immune responses typically requires sophisticated designs, such as nucleoside‐modified mRNA and optimized LNP formulations [190, 191]. For chronic disease management, such as repeated dosing for metabolic disorders or autoimmune conditions, high tolerability and anti‐inflammatory effects with minimal toxicity are paramount, provided that delivery efficiency is not compromised [144, 192]. Here, rapidly biodegradable lipids or synergistic formulations may be selected to enable repeated administration while maintaining efficacy, despite added formulation complexity, and long‐term stability must be rigorously tested. For targeted gene therapy, such as liver or lung delivery, targeting specificity takes precedence, and cholesterol modifications or SORT technology offer excellent organ selectivity, though the risk of reduced endosomal escape must be mitigated by carefully balancing the pKa value of ionizable lipids [193, 194]. For in vivo gene editing or systemic delivery of CRISPR components, the safety of the delivery system is often more critical than its large‐scale production capacity. Based on existing research, biomimetic or EV‐based systems can provide superior safety profiles, even though manufacturing challenges persist [195, 196]. In practice, a hybrid approach often yields the optimal outcome. For example, combining moderate charge reduction to lower inflammation with an optimized ionizable lipid to preserve endosomal escape, and a small amount of an endogenous anti‐inflammatory lipid to suppress residual innate immunity can achieve a favorable balance. High‐throughput screening, guided by AI predictions, can rapidly map the multi‐parameter design space. Ultimately, the “optimal” LNP is not the one that maximizes any single dimension, but rather the one that achieves the required therapeutic index, balancing efficacy, safety, and manufacturability for a specific disease indication.

### 4.6 Translational and Manufacturing Challenges

While the strategies outlined above, ranging from biomimetic coatings and anti‐inflammatory co‐delivery to complex lipid modifications, have demonstrated considerable potential in preclinical models, their clinical translation hinges on overcoming substantial challenges in chemistry, manufacturing, and control (CMC). These hurdles are often overlooked in mechanistic studies, yet they critically determine the feasibility, scalability, and regulatory approval pathway of LNP‐based therapeutics.

#### 4.6.1 Scalability and Reproducibility of Biomimetic Systems

Cell membrane‐derived biomimetic LNPs (e.g., erythrocyte, macrophage, or platelet membrane coatings) and EV‐based platforms offer unique advantages in immune evasion and targeted delivery. However, their clinical translation is hampered by several CMC challenges. First, sourcing and large‐scale production of cell membranes or EVs require robust cell culture systems, stringent quality control to ensure batch‐to‐batch consistency, and efficient purification methods to remove cellular contaminants and guarantee sterility [195]. Second, the integration of biological membranes with synthetic LNPs introduces additional complexity in formulation stability, as membrane proteins may denature during storage or upon exposure to shear forces during manufacturing [197]. Third, the regulatory classification of such hybrid systems remains ambiguous. Depending on their composition and mechanism of action, they may be categorized as biologics, combination products, or advanced therapy medicinal products, each with distinct approval pathways and manufacturing requirements that current regulatory frameworks are not yet fully equipped to address [198].

#### 4.6.2 Formulation Complexity for Co‐Delivery

Co‐delivery of nucleic acids with anti‐inflammatory agents (e.g., dexamethasone) in the same LNP presents a compelling strategy to mitigate inflammation while preserving therapeutic efficacy. However, this approach introduces several formulation challenges. The physicochemical compatibility between nucleic acids and immunomodulatory agents must be carefully balanced, as differences in hydrophobicity, charge, and molecular weight can affect encapsulation efficiency, particle size distribution, and long‐term stability [178, 199, 200]. Furthermore, the pharmacokinetic profiles of the two payloads may differ substantially. For instance, small‐molecule anti‐inflammatory drugs may be rapidly released from LNPs, while nucleic acids require sustained intracellular delivery to exert their effect. This discrepancy can lead to a temporal mismatch in pharmacodynamic activity [200]. Achieving coordinated release of both payloads at the target site remains a non‐trivial formulation challenge.

#### 4.6.3 Chemical Complexity and Supply Chain Limitations

The development of novel ionizable lipids, biodegradable lipids, and functionalized PEG‐lipids has enabled precise modulation of LNP immunogenicity and organ targeting. However, the synthesis of these complex lipids often involves multi‐step organic synthesis, chiral purification, and stringent impurity profiling. The presence of reactive impurities (e.g., residual catalysts, byproducts) can induce unintended inflammatory responses or compromise product stability [201]. Additionally, the supply chain for such specialty lipids is often constrained, with few manufacturers capable of producing them under current Good Manufacturing Practice (GMP) conditions, posing scalability risks for late‐stage clinical development [201, 202]. Regulatory expectations for full characterization of lipid impurities and degradation products further add to the development burden.

#### 4.6.4 General CMC Considerations for LNP Products

Beyond strategy‐specific challenges, several general CMC hurdles are applicable to all LNP‐based therapeutics. Sterilization remains a critical concern, as LNPs are typically heat‐labile and cannot withstand terminal sterilization methods such as autoclaving. Aseptic processing under GMP is therefore required, which increases manufacturing complexity and costs [1]. The long‐term stability of LNP formulations, particularly those containing mRNA or other labile nucleic acids, often necessitates frozen storage (‐20°C or ‐80°C) to maintain potency, which poses challenges for distribution, supply chain logistics, and clinical adoption [201, 203]. Analytical methods for characterizing LNPs, such as particle size distribution, encapsulation efficiency, lipid composition, and nucleic acid integrity, must be validated to meet regulatory standards, yet standardized reference methods are still in the process of evolution [201, 204]. Finally, raw material variability, particularly for lipid components sourced from different suppliers, can significantly impact product quality and reproducibility, necessitating robust supplier qualification and raw material testing programs [201].

Collectively, these CMC challenges underscore the necessity of early integration of manufacturability considerations into LNP design. Strategies that demonstrate promise in small‐scale preclinical studies may not seamlessly translate to commercial‐scale production. Therefore, close collaboration among formulation scientists, manufacturing engineers, and regulatory experts is imperative to de‐risk the clinical translation of next‐generation LNP platforms.

## 5 Prospects and Conclusions

Inflammatory responses triggered by nucleic acid‐delivering LNPs embody a dual nature: while providing essential adjuvant potency for effective immunity, they transform into critical safety liabilities when exceeding thresholds of intensity, duration, or spatial distribution. This duality is particularly consequential for developing LNP‐based therapies against inflammation‐driven chronic diseases, dictating that future efforts prioritize not mere inflammation suppression, but precision immunomodulation through multidimensional systems strategies tailored to specific therapeutic contexts.

This context‐dependent duality is exemplified by vaccine applications, where the goal is not to eliminate inflammation but to harness it in a controlled manner. In mRNA vaccine formulations, the inherent immunostimulatory properties of LNPs, including activation of the TLR4 signaling and type I interferon responses, directly contribute to their adjuvant activity [41, 205]. Specifically, IL‐6 and IL‐1 signaling promote the differentiation of Tfh cells and the formation of germinal centers, both of which are essential for generating durable antibody responses [41, 205]. Meanwhile, type I interferons support CD8^+^ T cell expansion [40]. Rather than suppressing these signals, the design objective for vaccines is to achieve spatially and temporally controlled inflammatory cues that maximize immunogenicity while minimizing systemic reactogenicity (e.g., fever, fatigue). Recent advances in LNP engineering, such as optimizing ionizable lipid structure, substituting cholesterol with plant‐derived sterols, or incorporating biodegradable lipid tails, have demonstrated the feasibility of decoupling pro‐inflammatory signals from adjuvant activity. These modifications yield formulations with reduced reactogenicity while preserving robust immunogenicity [38, 59].

Conversely, for non‐vaccine applications, such as chronic disease management, repeated‐dose regimens, or gene therapy, uncontrolled inflammation emerges as a critical safety liability that must be mitigated. Current evidence indicates that LNP‐induced inflammation primarily stems from complex interactions between LNP components (e.g., charge characteristics of ionizable lipids, PEGylated lipids) and biological systems. During endosomal escape and related processes, LNPs can activate TLRs and other PRRs, triggering innate immune responses and proinflammatory cytokine release. This manifests clinically as transient reactions such as fever, erythema, and edema for LNP‐derived vaccines [177, 206, 207, 208, 209]. Notably, inflammatory responses vary significantly across LNP formulations, suggesting that component optimization, such as modulating ionizable lipid ratios or incorporating biodegradable motifs, can effectively balance delivery efficiency with immune activation [177, 210, 211]. However, the challenge becomes fundamentally systemic when pursuing extrahepatic targeting or complex delivery scenarios. For instance, introducing targeting ligands may enhance tissue specificity but concurrently alter protein corona composition and elevate complement activation risks [212, 213]. Consequently, any single‐parameter adjustment may cascade through multiple biological axes, necessitating design approaches grounded in systems integration principles.

From a clinical perspective, the safety challenges associated with LNPs manifest in three aspects: immunogenicity accumulation, off‐target organ toxicity, and individual variability in treatment response. Repeated administration may induce PEG antibody resistance and accelerate blood clearance, ultimately limiting long‐term therapeutic potential [206, 214]. Following systemic delivery, the predominant hepatic uptake of LNPs contrasts with the potential risks posed by accidental accumulation in sensitive organs such as the brain and heart, where unpredictable adverse effects could occur [215, 216]. Furthermore, interpatient differences in immune status and biological responses significantly affect the distribution and toxicity profiles of LNPs, necessitating the establishment of precise patient stratification strategies [70, 217]. The translational efficiency of LNP‐based drug development faces additional hurdles due to the inherent limitations of preclinical models. A fundamental challenge lies in the frequent disparity between in vitro performance and in vivo outcomes, where numerous nucleic acid delivery systems demonstrate robust cellular efficacy but fail in animal studies. This discrepancy arises because conventional in vitro models cannot adequately replicate the complex biological barriers and dynamic physiological conditions encountered in living organisms [218]. Compounding this issue, interspecies variations complicate data extrapolation. At the level of innate immune sensing, critical discrepancies exist: TLR8, which recognizes single‐stranded RNA and serves as a key sensor for mRNA‐LNPs in humans, is non‐functional in mice due to a species‐specific gene deletion, while mouse TLR7 exhibits hyper‐responsiveness to RNA motifs that may not elicit equivalent responses in human cells [219, 220]. Such interspecies mismatches imply that inflammatory responses observed in rodents may not accurately predict human reactivity. Adding another layer of complexity, complement system activity differs markedly across species. The pathways of complement activation (e.g., alternative versus lectin) and the efficiency of NP opsonization vary substantially between humans and rodents, with human sera often showing lower or qualitatively different C3 deposition compared to mouse sera [221]. Consequently, CARPA, a common infusion reaction to nanomedicines in humans, is poorly recapitulated in conventional mouse strains. These strains lack pre‐existing anti‐PEG antibodies and exhibit distinct complement system activity, rendering them largely insensitive to preclinical safety screening [110, 114]. Further complicating matters, inflammatory signaling thresholds also diverge. Following innate immune stimulation, mice display a markedly higher induction of the IL‐1 receptor antagonist relative to IL‐1 compared with humans. This imbalance dampens IL‐1 signaling and confers resistance to systemic inflammatory responses, potentially leading to an underestimation of cytokine release syndrome risks observed in clinical trials [222]. Beyond these immunological disparities, requirements for physicochemical optimization also vary across species. For example, non‐human primates and rodents demand distinct particle sizes (50–60 nm vs. 70–80 nm, respectively) and surface chemistry profiles, with non‐human primates typically requiring twice the PEG‐lipid content to achieve comparable delivery efficiency [223].

From a perspective of therapeutic scenario management, LNP‐induced inflammation presents both significant pharmacological potential and notable safety limitations. Efficacy fundamentally hinges on achieving a dynamic, context‐dependent immune balance: appropriately eliciting immune responses for vaccines, while minimizing nonspecific inflammation in gene editing or chronic inflammatory disease treatment. This precise balance between immunostimulation and immune tolerance is crucial for optimizing therapeutic benefits and mitigating adverse effects, thereby enabling the versatile application of LNPs across diverse scenarios like vaccines, gene editing‐based therapies, and chronic disease management.

Advancements in LNP technology, spanning targeted delivery systems, patient‐customized formulations, and combination therapies, are reshaping immunomodulatory paradigms. To fully realize this potential, future research should prioritize correlating multi‐omics profiles with clinical response patterns to establish intelligent design frameworks. This will enable the development of “context‐adaptive” LNP platforms capable of real‐time immune modulation calibrated to specific disease states and patient characteristics. Such innovations will not only accelerate clinical translation of nucleic acid‐loaded LNPs for inflammatory diseases and immune‐oncology applications but also facilitate the convergence of precision immunomodulation with practical therapeutic implementation.

## Author Contributions

R.M.H., W.H.S., and J.X.Z. contributed to the writing and editing of this manuscript. Y.D., C.W.L., Y.Y.M., and H.Y.O. researched data for the article and contributed to the discussion of content and writing. R.M.H., W.H.S., and J.X.Z. contributed to the discussions and writing, reviewing, and editing of the manuscript.

## Conflicts of Interest

The authors declare no conflicts of interest.

## Data Availability

The data that support the findings of this study are available from the corresponding author upon reasonable request.
